# How Not to Fall for the White Bear: Combined Frequency and Recency Manipulations Diminish Negation Effects on Overt Behavior

**DOI:** 10.5334/joc.62

**Published:** 2019-04-23

**Authors:** Robert Wirth, Wilfried Kunde, Roland Pfister

**Affiliations:** 1 Department of Psychology, Julius-Maximilians-University of Würzburg, Röntgenring, Würzburg, DE

**Keywords:** Cognitive Control, Action, Working memory

## Abstract

Processing negated mental representations comes with a price: Not only are negations harder to resolve than affirmative statements, but they may even invoke ironic effects, producing the exact opposite of the intended outcome. Negation effects also behave ironically when subjected to high-frequency training; when they are confronted often, the difficulty to process negations strangely increases. Here we show that negation effects can be mitigated under certain circumstances. Based on models of cognitive control and conflict adaptation, we hypothesized that negation effects diminish when two criteria are met: negations have to be resolved not only frequently, but also just recently. We confirmed this prediction in two experiments by using an innovative, two-dimensional finger tracking design, in which we measured the influence of the original semantic content during negation processing via temporal and spatial measures. Negation effects were present throughout the experiment, but were reduced after recent negations, particularly during or after a high-frequency negation context. The combined influence of frequency and recency thus seems to be the most successful and promising attempt to mitigate ironic negation effects on overt behavior.

## Introduction

“Don’t use no double negatives”, states a classic guideline on scientific writing (Trigg, 1979). And rightfully so: Already a single negation requires effortful cognitive processing that may not only fail to reach the intended outcome, but may even produce the exact opposite result. Such detrimental effects of negations are also known as the white bear effect, which lends its name to findings that participants who actively tried not to think of a white bear actually found themselves to be haunted by precisely this mental image (Wegner, Schneider, Carter, & White, 1987).

Previous attempts to counter the detrimental effects of negation processing via cognitive training interventions came with only limited success (Gawronski, Deutsch, Mbirkou, Seibt, & Strack, 2008). Here we propose a novel approach to countering negation effects that is rooted in theoretical models on cognitive control, and we test this proposal in two high-powered experiments. To motivate this approach, we will first outline the current state of the literature on negation processing and conclude with a brief summary on theoretical and empirical developments in research on cognitive control.

### Negation processing

Negation effects have often been reported in experiments on thought suppression, which bear direct implications for real-world behavior. Imagine a college student starting up their computer to do coursework. If it was not for the coursework, the student might be inclined to read through recent posts on social media pages, but this behavior would jeopardize any coursework-related plans. At first sight, it seems as if an explicit implementation intention (“*if the computer has started, I will not visit Facebook”*) might help the student (Gollwitzer & Sheeran, 2006). However, research suggests that holding this intention may, in fact, increase the student’s likelihood of falling back to the unwanted habit (Adriaanse, Van Oosten, De Ridder, De Wit, & Evers, 2011). That is: Explicitly intending not to do something may at times promote a mental representation of precisely the unwanted behavior (Gollwitzer & Oettingen, 2012).

The student seeking to overcome an unwanted habit would therefore be well-advised to employ different strategies, such as replacing the habit with a more desirable behavior (“*if the computer has started, I will immediately start my word processor*”) or simply intending to ignore habit-related cues (“*if the computer has started, I will ignore the Facebook icon*”). These strategies might be especially promising, because strategies such as ignoring certain stimuli can be trained to further improve performance (Cunningham & Egeth, 2016).

There might be situations, however, in which negation is the only sensible option. For instance, certain stereotypes may not be easily ignored or countered by wanted behavior. These situations pose a considerable challenge, because even extended training of stereotype negation has been shown to enhance rather than reduce stereotyping (Gawronski et al., 2008). These findings suggest that, in contrast to deliberate ignoring (Cunningham & Egeth, 2016), the cognitive requirements of negation processing cannot be mitigated easily by high-frequency training.

Theoretical models of how negations are represented seem to agree with this notion (Gilbert, 1991; Wegner, 2009). Intuitively, one might follow the *Cartesian approach*, which assumes that mental representations are managed by two separate and serial processes: *Comprehension* recollects the pure semantic content of a representation, followed by an *assessment* of the semantic content as true or false. However, empirical evidence suggests that the human mind is better described by the *Spinozan approach* (Gilbert, 1991; Gilbert, Krull, Malone, 1990; Wegner, Coulton, & Wentzlaff, 1985). In this view, the comprehension of an idea inherently entails that its semantic content is accepted as true. Comprehension and assessment are conceptualized in a single, joint process. False statements therefore require an additional process that rejects the idea and relabels the automatically accepted content as false (for possible limitations and extensions, see Hasson, Simmons, & Todorov, 2005).

Evidence for this theoretical conceptualization comes from research that employed so-called innuendo procedures in which participants receive descriptive statements regarding a target person before rating their impression of the described person (Wegner, Wenzlaff, Kerker, & Beattie, 1981).^1^ When the descriptions included a negation of a negative attribute, e.g., “Bob Talbert is not linked to Mafia”, participants consistently gave less favorable ratings compared to a neutral baseline (e.g., “Bob Talbert arrived in City”). Furthermore, such innuendo effects were found to be particularly pervasive when participants were interrupted mid-way during processing of the statements (Gilbert et al., 1990). The unacceptance of an idea thus seems to require time and cognitive effort, and as the semantic content of a negation is always automatically accepted in a first step, this unacceptance process is required for every single instance of a negation, irrespective of its frequency. Therefore, high-frequency training of negations alone can hardly reduce their ironic effects (Gawronski et al., 2008).

So far, it seems as if the processing of negations is difficult and, ironically, attempts to mitigate negation effects may even result in the complete opposite. However, we propose that a potent strategy to counter negation effects emerges when negation processing is viewed from the perspective of conflict processing and cognitive control (Schroder, Moran, Moser, & Altmann, 2012; for an early proposal, see Seymour, 1977).

### Cognitive Control

For the case of negation processing, we argue that the concept of cognitive conflict and its resolution is especially promising because of structural similarities of how negations and cognitive conflict are processed: The resolution of a negation involves two competing representations, one of which is activated automatically (the semantic content of the negated information) while the other (the negation of that content itself) requires effortful processing. This process mirrors the resolution of conflict induced by tasks that require the inhibition of a prepotent response (such as incongruent Stroop stimuli or unexpected NoGo stimuli). Recent evidence indeed suggests negations to rely on precisely this type of response inhibition (de Vega et al., 2016; Dudschig & Kaup, 2018).

On a theoretical level, it is commonly assumed that conflict adaptation comprises two separate mechanisms: Conflict monitoring mechanisms that detect the presence of competing action plans in the first place, and cognitive control mechanisms that adjust attentional control states (Botvinick, Braver, Barch, Carter, & Cohen, 2001; Botvinick, Cohen & Carter, 2004). Such control states, in turn, can then help to resolve upcoming conflict (Egner, 2007; Hubbard, Kuhns, Schäfer, & Mayr, 2017). Based on this theoretical framework, the literature on cognitive conflict and cognitive control offers two clear methods to reduce cognitive conflict. First, an expected high likelihood of conflicting events due to increasing their list-wide frequency is thought to diminish conflict effects (Funes, Lupianez & Humphreys, 2010; Logan & Zbrodoff, 1979). Second, experiencing a cognitively demanding event in a very recent (typically the last) trial has also been shown to minimize the impact of the conflicting information (e.g., Botvinick, Braver, Barch, Carter, & Cohen, 2001; Botvinick, Cohen & Carter, 2004; Gratton, Coles, & Donchin, 1992; Hubbard et al., 2017). Furthermore, positive effects of conflict frequency may at least partly be explained as being due to a higher probability of benefits due to conflict recency (Botvinick et al., 2001; Torres-Quesada, Lupiáñez, Milliken, & Funes, 2014). Acknowledging a crucial role of conflict recency, in turn, offers a promising approach to mitigating the detrimental effects of negation processing as we will argue in the following. With respect to negation processing, the evidence suggests that control of negation costs as a consequence of overall negation frequency is in fact not possible (Gawronski et al., 2008), and also control based on recent events alone might be difficult to establish (Wirth, Pfister, Foerster, Huestegge, & Kunde, 2016). A possible interplay of both factors, by contrast, holds promise for expediting negation processing and will be tested in the following experiments.

### The present study

The research on cognitive conflict and control reviewed above suggests that previous attempts to mitigate negation effects might have fell just short of providing a powerful solution: The costs of negation processing might be reduced if negations are not only experienced frequently (as in previous training studies; Gawronski et al., 2008) but rather if a particular negation has been processed both, frequently and very recently. Such an impact of recency is also conceivable within the *Spinozan model* of negation processing: When a mental representation has been recollected and negated, repeating that same process for a subsequent negation shortly after might not be necessary, because traces of the negated representation might still be in working memory and can be used rather than recollecting and rejecting the mental representation’s semantic content anew.

## Experiment 1

We set out to investigate the impact of frequency and recency on negation effects in a combined design by confronting participants either with a high or low frequency of negations and analyzing the impact of negation frequency and recency on the costs incurred by negation processing.

Because typical innuendo procedures as described in the introduction (Gilbert et al., 1990; Wegner et al., 1985) provide a rather indirect take on negation processing via memory failures for interrupted and non-interrupted items, we chose to adopt a method that allows for a more direct way of capturing the processing costs of negations. Such procedures have recently been promoted in research on psycholinguistics as well as in speeded choice reaction tasks. A typical design from psycholinguistics revolves around a sentence verification task in which participants are given true or false statements that may or may not contain a negation (e.g., “Mice are not large” for a true statement containing a negation). Verifying a negated sentence typically takes longer than verifying a sentence without negation, and this effect on response times has long been taken as a direct measure of processing costs (Just & Carpenter, 1976; Wason, 1959; Wason & Johnson-Laird, 1972). Further highlighting the difficulty of resolving negations, electrophysiological markers of sentence and scene comprehension such as the N400 are unaffected by negation operators (Dudschig, Mackenzie, Leuthold, & Kaup, 2018).^2^ An even more direct measure of negation processing, however, emerges when participants are asked to respond via mouse- or finger-movements rather than keypresses, moving the mouse or their finger to one of two spatial locations as a response. Corresponding movement trajectory analyses have recently been shown to offer a unique and powerful window on the resolution of cognitive conflict (Calderon, Verguts, & Gevers, 2015; Dshemuchadse, Scherbaum, & Goschke, 2013), and the trajectories of such movements have indeed been shown to yield a spatial measure for an initial activation of the non-negated response (Dale & Duran, 2011).

Furthermore, movement trajectories may at times be a more sensitive measure of negation processing compared to reaction times, as suggested by a study that employed a simple choice-reaction task (Wirth et al., 2016). In this study, we asked participants to respond to stimuli by diagonal swiping movements (left or right) on an iPad screen, and a mapping rule specified the stimulus-response mapping. Participants were cued in each trial whether to use the original mapping rule or whether they were to negate this rule and perform the opposite movement.^3^ In this setting, negations incurred a pronounced attraction of the movement trajectory toward the opposite target area, i.e., towards the target that would have been indicated by the non-negated (read: original) rule. Critically, this effect was present to the same extent when two negations were performed directly after each other, i.e., there was no sequential adaptation effect on the trajectory data. Yet, when participants were instructed with two separate opposing stimulus-response rules as compared to a single rule that had to be negated, the use of the opposing rule came with improved movement execution when having employed that rule also in the trial before. So, apparently the repetition of a negation alone does not suffice to improve performance, whereas the repetition of an opposing rule does improve performance.

We conjecture that repetition benefits for negations (i.e., recency effects) might emerge, however, if the need for control is emphasized. So we speculate that having experienced negations often may not reduce negation effects overall, as has been found before, but a high frequency of negations may signal a higher need for control, which presumably might trigger adaptation based on recent negations, allowing for negation repetition benefits. Further, we expect that if this need for control has been signaled once (i.e., once a high frequency of negations has been experienced), only then recency effects might emerge, suggesting that the group that starts with the low frequency of negations, recency effects should only emerge once the frequency of negations is increased, whereas the group that starts with the high frequency should benefit from recent negations from the start, and this might even carry over to the later blocks with a lower frequency (for a similar argument, see Wirth et al., 2018).

Because of the high sensitivity of movement trajectories to cognitive conflict in general (Erb, Moher, Song, & Sobel, 2017; Calderon et al., 2015; Dshemuchadse et al., 2013, Scherbaum, Dshemuchadse, & Fisher, 2010; Kieslich & Hilbig, 2014; Song & Nakayama, 2009) and to negation processing in particular (Dale & Duran, 2011; Wirth et al., 2016) we chose to address the potential impact of negation recency and negation frequency on movement trajectories in a choice reaction task. We expected these movement trajectory measures to yield strong negation costs but, crucially, negation costs should be reduced or even absent if participants could benefit from both, frequent and recent negation processing.

### Methods

In both experiments that are presented here, we report how we determined our sample size, all data exclusions (if any), all manipulations, and all measures in the study (Simmons, Nelson, & Simonsohn, 2012).

#### Participants

Eighty participants were recruited (mean age = 25.5 years, SD = 4.7, 28 male, 8 left-handed) and received either course credit or €10 monetary compensation. For power analyses, we first considered the effect of negation processing on movement trajectories, which has been reported to be sizeable (e.g., *d*_z_ = 1.30 for Exp. 3 in Wirth et al., 2016). Reliable estimates for the effects of negation frequency and recency on these negation costs were not available in the literature while planning this study so that we decided to recruit sufficient participants to detect a medium-sized effect of *d*_z_ = 0.50 with high power (1-β = .99), while at the same time providing ample chances to detect even smaller effects (1-β ≥ .8 for *d*_z_ > 0.32).

All participants gave informed consent, were naïve to the purpose of the experiment and were debriefed after the session. The data of one participant was removed due to technical difficulties during testing, and five participants were removed from the sample due to high error rates (>25%, computed as (number of errors + omissions)/number of all trials).

#### Apparatus and stimuli

**Figure 1:**
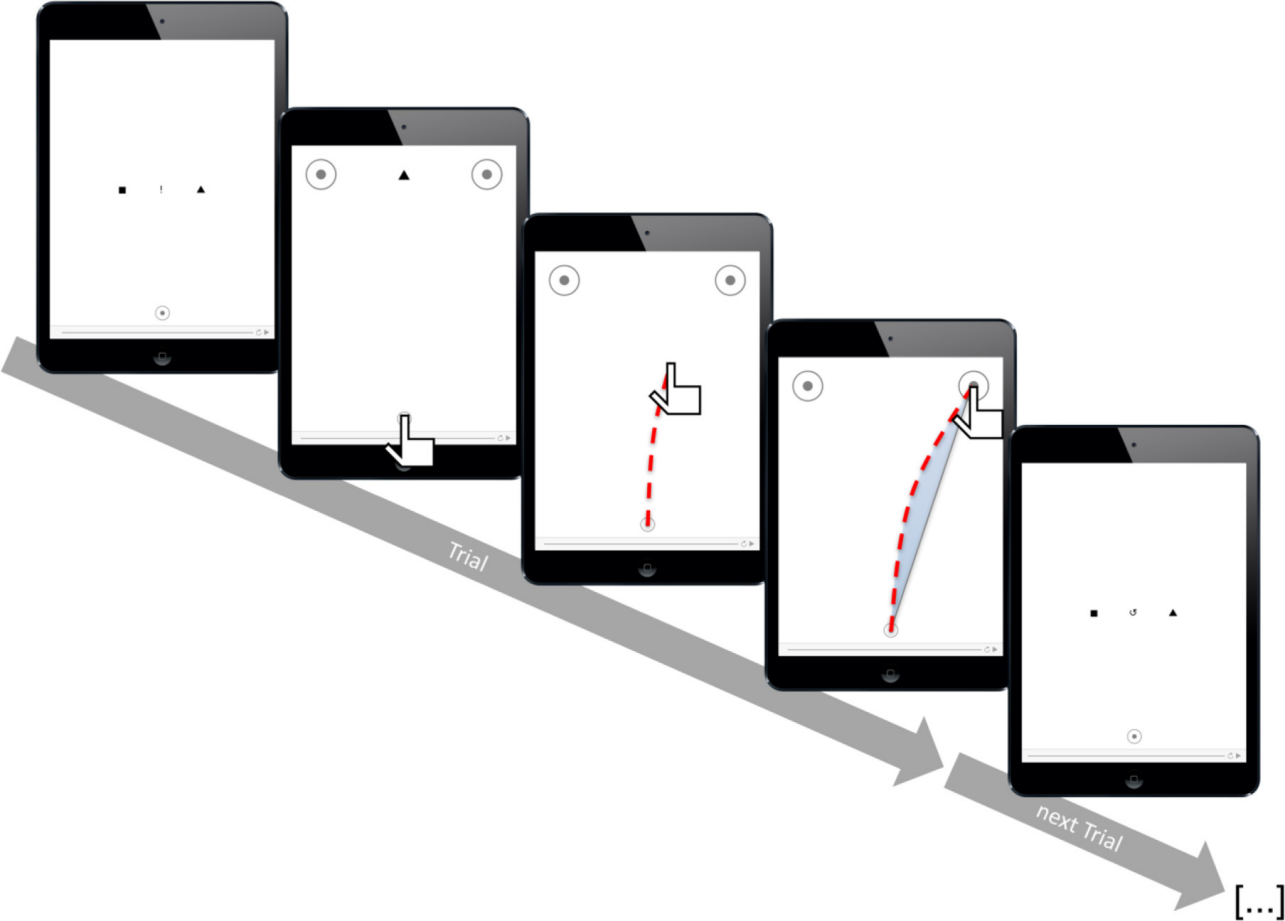
**Procedure of the experiments.** Before each trial, participants were reminded of the mapping rule, together with the instruction to either perform a standard response according to the displayed mapping rule or to negate this mapping rule in the next trial. As soon as participants put their finger on the starting area, the mapping rule disappeared, and the two target areas and the target symbol appeared, prompting movements to the left or the right. The target symbol disappeared when the finger left the starting area. A trial was completed when the finger was lifted from the screen inside one of the two target areas, and the next trial started immediately with the corresponding standard or negation instructions. The stimuli shown here are taken from Experiment 1, for Experiment 2, the mapping rule (first and last screen) would consist of male and female symbols (♂/♀) rather than square and triangle, and the stimulus in the second screen would be replaced with a photo of a male or a female face.

The experiment was run on an iPad 2 (screen resolution of 786 × 1024 px) in portrait mode with a viewing distance of about 50 cm. Participants used the index finger of their dominant hand to operate the touchscreen, which sampled the finger movements at 100 Hz. We used two shapes (■/▲) as stimuli to prompt movements to the left or to the right target area (two circles of 2 cm in diameter in the upper left and right corners of the display). The target areas were separated by 11 cm (center-to-center) and were displayed against a white background. The starting position for the movement (a circle of 1 cm in diameter) was located at the bottom center of the screen, 17 cm from the middle of the two target positions at an angle of 31° to each side (Figure 1).

In between trials, the two stimulus shapes were displayed to the left and right of the screen center to remind participants of the stimulus-response mapping. In between the two shapes, an exclamation mark (!) instructed standard responses based on the displayed mapping rule, a circular arrow (↺) prompted participants to negate the displayed mapping.

#### Procedure

Before each trial, the stimulus-response mapping appeared on screen, together with the instruction cue to perform either a standard response or a negation response, which varied randomly from trial to trial. In standard trials, the shape that was displayed on the left side would indicate that a movement to the left had to be executed, and the shape on the right side was associated with movements to the right. In negation trials, this displayed mapping rule had to be negated; the response that a stimulus originally required was now contraindicated: In particular, the shape on the left side of the screen now indicated *not* to execute a movement to the left, but instead move to the right, and the shape on the right side indicated *not* to execute a movement to the right, but rather to the left. Participants started a trial by touching the starting area with the index finger of the dominant hand. Immediately, the display was cleared and one of the two stimulus shapes appeared between the two target areas to indicate whether a movement to the left or a movement to the right had to be executed. Half of the participants were instructed to make a smooth finger movement to the left target area in response to a square and to the right target area in response to a triangle (cf. Figure 1). The other half of the participants was instructed with the opposite mapping for counterbalancing. The stimulus symbol disappeared as soon as the finger left the starting area. A trial ended when the finger was lifted from the touchscreen. Error feedback was displayed if participants reached the wrong target area or failed to hit one of the designated target areas at all.

The proportion of negation trials was manipulated between experimental halves: in blocks with a low proportion of negations (low-PN), the displayed mapping rule had to be negated in one out of four trials. In blocks with a high proportion of negations (high-PN), the mapping rule had to be negated in three out of four trials. The proportion of negations changed after half of the experimental blocks, and the order of presentation (first half: low-PN, second half: high-PN vs. first half: high-PN, second half: low-PN) was counterbalanced between participants. The PN-manipulation was not communicated via instructions, so participants had to experience the changing frequency. To remove any (re)learning- and adjustment effects, we will consider the first block of each PN condition as practice and exclude it from further analysis.

Instructions stressed that responses had to be delivered quickly and accurately; still the experiment was self-paced, so participants chose on their own when to start a trial and how long they took breaks in between blocks. Participants completed 20 blocks of 64 trials each. An experimental session lasted about 1 hour.

### Results

#### Preprocessing

We measured three variables of each movement: The time it took participants to leave the starting area after touching it (initiation time; IT), the duration of the movement, from leaving the starting area until lifting the finger from the screen (movement time; MT), and the area between the actual movement trajectory and a straight line from start- to endpoint (area under the curve; AUC; shaded area in Figure 1). AUC was computed from the time-normalized coordinate data of each trial by using custom MATLAB scripts (The Mathworks, Inc.). Movements to the left were mirrored at the vertical midline for all analyses. AUC was computed as the signed area relative to a straight line from start- to endpoint of the movement. Positive values indicate attraction toward the opposite side (indicating a persisting influence of the standard mapping rule in case of negations), negative values indicate attraction toward the nearest edge of the display.

#### Data selection and analyses

For all analyses, the first block of each PN condition was considered practice and removed.^4^ We then omitted trials in which participants failed to act according to the instruction or failed to hit any of the two target areas at all (6.0%) and trials following errors (5.1%). Trials were discarded as outliers if any of the measures (IT, MT, AUC) deviated more than 2.5 standard deviations from the participants’ individual cell mean (5.3%). Data for each DV was then aggregated separately for each participant and each combination of PN, preceding response type, and current response type. To keep it frugal, we restrict the main analyses on the costs incurred by negations relative to standard responses (Δ = current negation minus current standard, computed separately for the aggregate values of each participant and each combination of PN and preceding response type). The full analysis of every measure, including the factor current response type, as well as the corresponding raw data, can be found in the Supplementary Material online (www.osf.io/kzwp6, page 2).

Mean ΔITs, ΔMTs, and ΔAUCs were analyzed in a 2 × 2 × 2 analysis of variance (ANOVA) with preceding response type (standard vs. negation) and proportion negation (low-PN vs. high-PN) as within-subject factors, and proportion order (low-PN-first vs. high-PN-first) as a between-subjects factor. Because movement trajectories, as measured via AUCs, are particularly sensitive to negation effects (Dale & Duran, 2011; Wirth et al., 2016), we will consider negation costs to be mitigated when negation effects are absent (or reversed) mainly for AUCs, but we also report the temporal DVs for completeness. Based on models of cognitive control, we expect an overall effect of recency (reduction of negation effects after a negation response compared to after a standard response), and these recency effects should be larger with a high PN, which should manifest in an interaction between preceding response type and proportion negation. Controlling for the order of proportion negation, we expect this interaction to be especially pronounced in the low-PN-first group.

#### ΔITs

**Figure 2:**
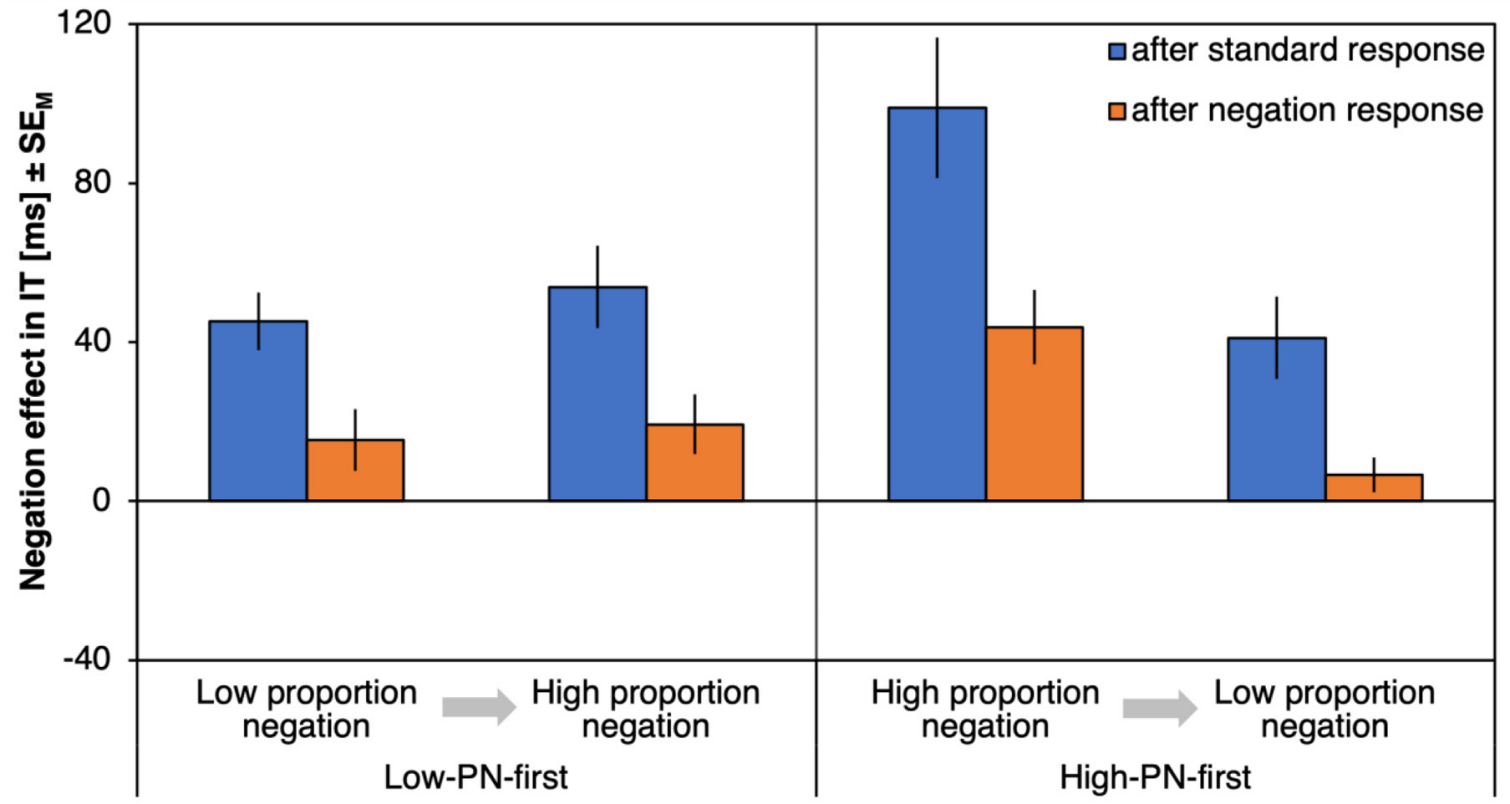
**Results of Experiment 1, initiation times.** Negation effects on initiation times (ΔIT), plotted as a function of proportion negation (PN) and proportion order (abscissa) and preceding response type (left, blue bars for trials following standard responses; right, orange bars for trials following negation responses). Error bars represent standard errors of the mean.

Figure 2 shows the negation effects on ITs (ΔIT) as a function of preceding response type, proportion negation and proportion order. There was a significant effect of preceding response type, *F*(1,72) = 24.27, *p* < .001, η_p_^2^ = .25, with stronger negation effects after a standard response (59 ms) compared to after a negation response (21ms). Negation effects were especially reduced after negation responses (compared to after a standard response) for the high-PN condition (with a reduction of ΔIT by 45 ms) relative to the low-PN condition (with a reduction of 32ms) as indicated by an interaction of preceding response type and proportion negation, *F*(1,72) = 4.62, *p* = .035, η_p_^2^ = .06. Proportion order further interacted with proportion negation, *F*(1,72) = 13.03, *p* = .001, η_p_^2^ = .15, with smaller negation effects in low-PN relative to high-PN blocks for participants who started with the high-PN condition (with a difference between ΔITs of 48 ms) compared to those who started with the low-PN condition (with a difference of 6 ms). A main effect of proportion negation, *F*(1,72) = 22.34, *p* < .001, η_p_^2^ = .24, further indicated smaller negation effects in the low-PN blocks (27ms) relative to the high-PN blocks (53 ms). No other effects were significant, *F*s < 2.08, *p*s > .154 (for an overview, see Table 1).

**Table 1:** **Results of Experiment 1.** Overview of the ANOVA results of Experiment 1 for initiation times (ITs), movement times (MTs) and areas under the curve (AUCs). All analyses were run on the negation effects in each design cell (i.e., current negation minus current standard).

	Effect	*F*	*p*	η_p_^2^

**IT**	preceding response type (N-1)	24.27	<.001	.25
	proportion negation (PN)	22.34	<.001	.24
	proportion order (PO)	2.08	.154	.03
	N-1 × PN	4,62	.035	.06
	N-1 × PO	<1.00	.424	.01
	PN × PO	13.03	.001	.15
	N-1 × PN × PO	1.79	.185	.02

**MT**	preceding response type (N-1)	68.33	<.001	.49
	proportion negation (PN)	24.11	<.001	.25
	proportion order (PO)	1.36	.248	.02
	N-1 × PN	4.59	.035	.06
	N-1 × PO	<1.00	.920	<.01
	PN × PO	<1.00	.784	<.01
	N-1 × PN × PO	4.24	.043	.06

**AUC**	preceding response type (N-1)	53.25	<.001	.43
	proportion negation (PN)	1.94	.168	.03
	proportion order (PO)	<1.00	.636	<.01
	N-1 × PN	6.82	.011	.09
	N-1 × PO	1.54	.219	.02
	PN × PO	4.10	.047	.05
	N-1 × PN × PO	7.41	.008	.09

Response costs for negations after standard responses differed from zero for all combinations of proportion negation and proportion order, |*t*|s > 3.94, *p*s < .001, *d*s > 0.67 (Figure 2, blue bars), whereas response costs for repeated negations differed from zero only in high-PN blocks, |*t*|s > 2.57, *p*s < .014, *d*s > 0.42, but not in the low-PN blocks, |*t*|s < 1.99, *p*s > .054, *d*s < 0.33 (Figure 2, orange bars).

#### ΔMTs

**Figure 3:**
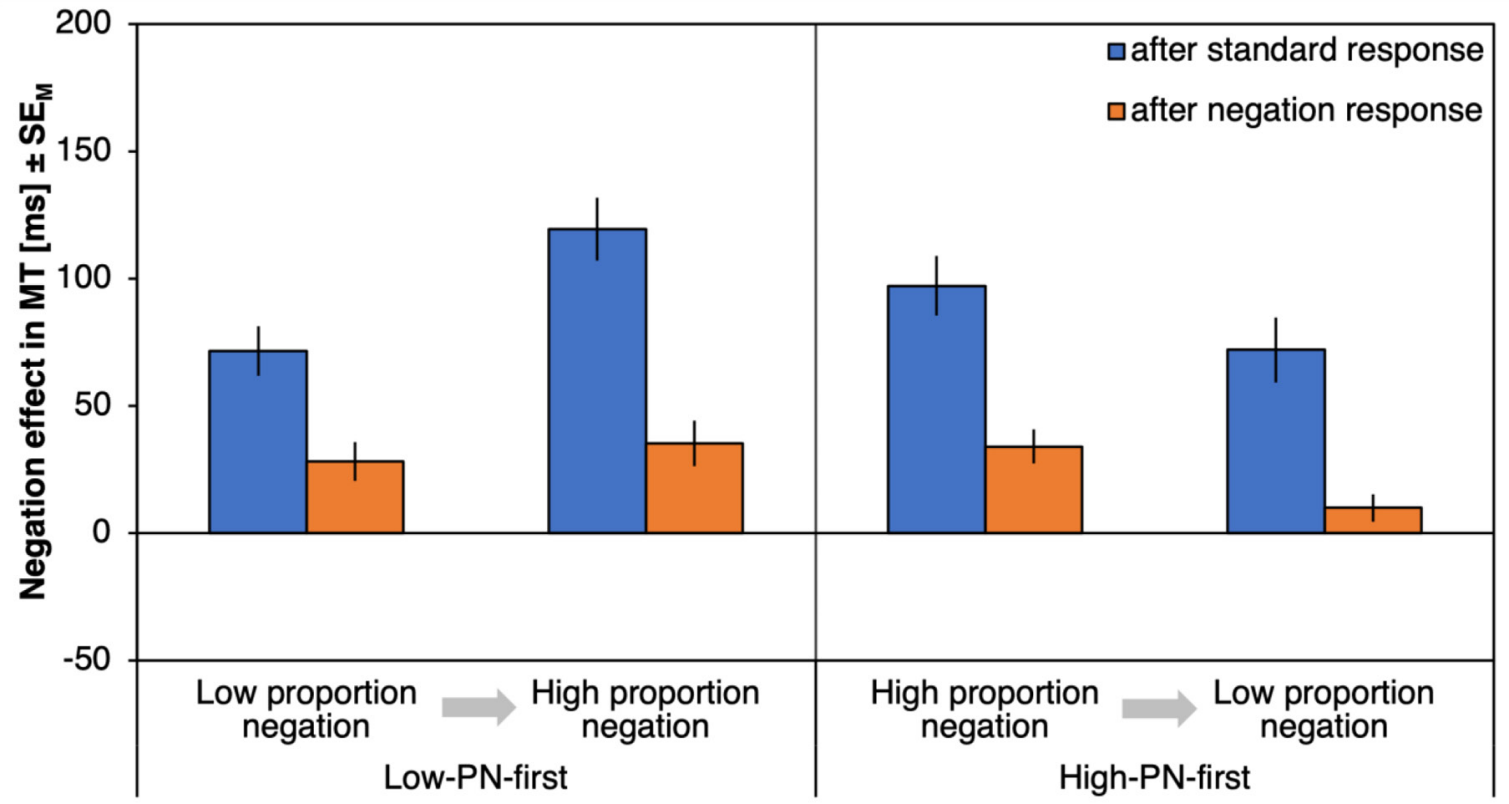
**Results of Experiment 1, movement times.** Negation effects on movement times (ΔMT), plotted as a function of proportion negation and proportion order (abscissa) and preceding response type (left, blue bars for trials following standard responses; right, orange bars for trials following negation responses). Error bars represent standard errors of the mean.

Figure 3 shows the negation effects on MTs (ΔMT) as a function of preceding response type, proportion negation and proportion order. There was a significant effect of preceding response type, *F*(1,72) = 68.33, *p* < .001, η_p_^2^ = .49, with stronger negation effects after a standard response (90 ms) compared to after a negation response (27 ms). Negation effects were especially reduced after negation responses (compared to after standard responses) for the high-PN condition (with a reduction of ΔMT by 74 ms) relative to the low-PN condition (with a reduction of 53 ms) as indicated by an interaction of preceding response type and proportion negation, *F*(1,72) = 4.59, *p* = .035, η_p_^2^ = .06. A main effect of proportion negation, *F*(1,72) = 24.11, *p* < .001, η_p_^2^ = .25, further indicated larger negation effects in the high-PN blocks (72 ms) relative to the low-PN blocks (45 ms). Finally, the three-way interaction was significant, *F*(1,72) = 4.24, *p* = .043, η_p_^2^ = .06, qualifying a much smaller difference between the negation effects after standard and negation responses in the low-PN condition of the low-PN-first subgroup (Figure 3, leftmost bars, Δ = 44 ms) compared to the same subgroup in the high-PN condition (Δ = 85 ms), *t*(37) = 2.23, *p* = .031, *d* = 0.36, but no difference in any comparison involving the high-PN-first subgroup, |*t*|s > 1.24, *p*s > .222. No other effects were significant, *F*s < 1.36, *p*s > .248 (for an overview, see Table 1).

Response costs for negations after standard responses differed from zero for all combinations of proportion negation and proportion order, |*t*|s > 5.64, *p*s < .001, *d*s > 0.93 (Figure 3, blue bars), whereas response costs for repeated negations differed from zero for all combinations, |*t*|s > 3.64, *p*s < .001, *d*s > 0.60, except for the low-PN condition of the high-PN-first group, *t*(35) = 1.84, *p* = .074, *d* = 0.30 (Figure 3, orange bars).

#### ΔAUCs

**Figure 4:**
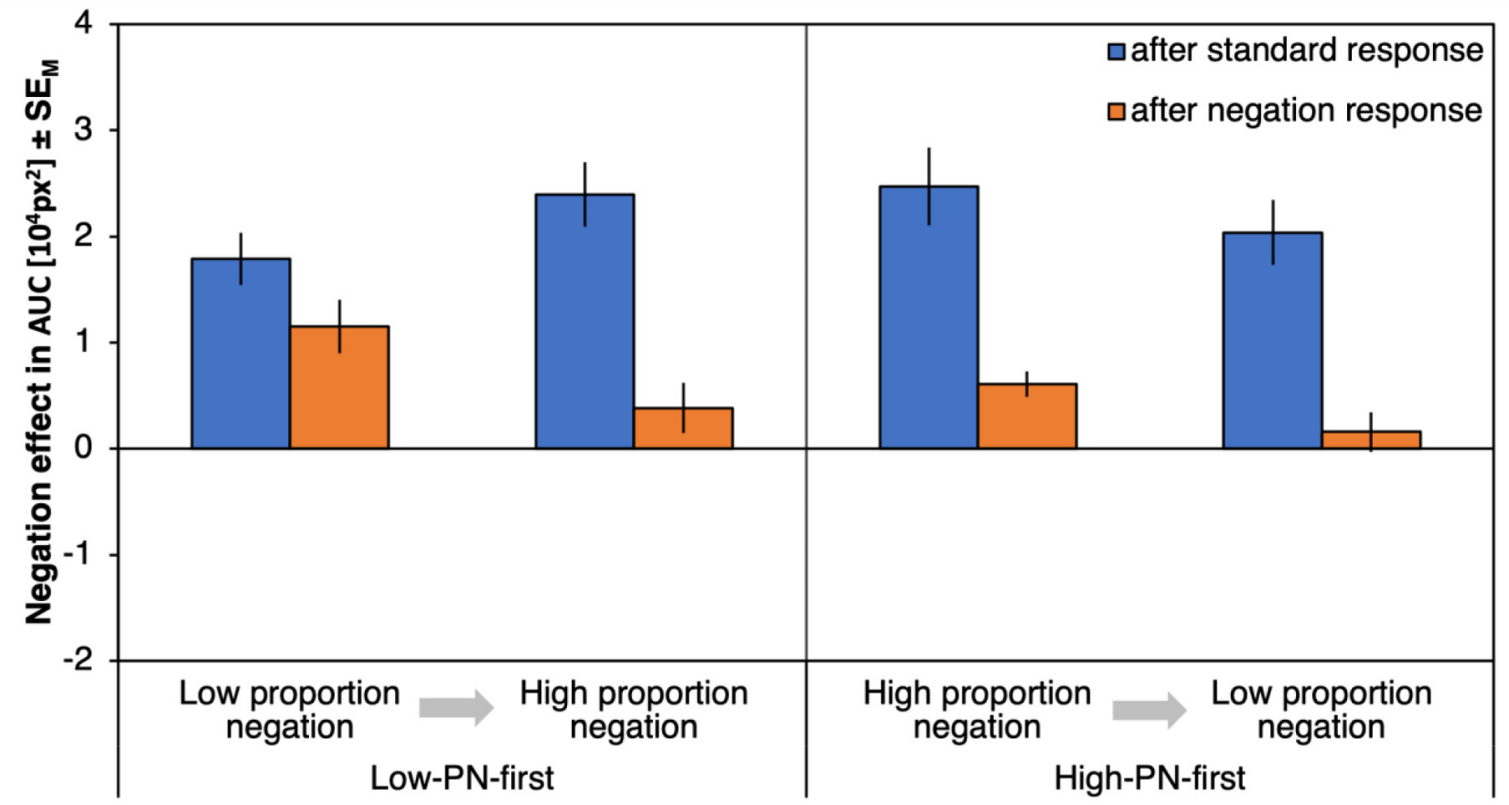
**Results of Experiment 1, areas under the curve.** Negation effects on areas under the curve (ΔAUC), plotted as a function of proportion negation and proportion order (abscissa) and preceding response type (left, blue bars for trials following standard responses; right, orange bars for trials following negation responses). Error bars represent standard errors of the mean.

Figure 4 shows the negation effects on AUCs (ΔAUC) as a function of preceding response type, proportion negation and proportion order. There was a significant effect of preceding response type, *F*(1,72) = 53.25, *p* < .001, η_p_^2^ = .43, with stronger negation effects after a standard response (21718 px^2^) compared to after a negation response (5747 px^2^). Negation effects were especially reduced after negation responses (compared to after standard responses) for the high-PN condition (with a reduction of ΔAUC by 19335 px^2^) relative to the low-PN condition (with a reduction of 12606 px^2^) as indicated by an interaction of preceding response type and proportion negation, *F*(1,72) = 6.82, *p* = .011, η_p_^2^ = .09. Proportion order further interacted with proportion negation, *F*(1,72) = 4.10, *p* = .047, η_p_^2^ = .05, with smaller negation effects in low-PN relative to high-PN blocks for participants who started with the high-PN condition (with a difference between ΔAUCs of 4395 px^2^) compared to those who started with the low-PN condition (with a difference of –810 px^2^). Finally, the three-way interaction was significant, *F*(1,72) = 7.41, *p* = .008, η_p_^2^ = .09, qualifying a much smaller difference between the negation effects after standard and negation responses in the low-PN condition of the low-PN-first subgroup (Figure 4, leftmost bars, Δ = 6385 px^2^) compared to any of the other conditions (Δs > 18543 px^2^). No other effects were significant, *F*s < 1.94, *p*s > .168 (for an overview, see Table 1).

Response costs for negations after standard responses differed from zero for all combinations of proportion negation and proportion order, |*t*|s > 6.68, *p*s < .001, *d*s > 1.11 (Figure 4, blue bars), whereas response costs for repeated negations differed from zero only for the first condition per proportion order, |*t*|s > 4.59, *p*s < .001, *d*s > 0.74, but not for the second, |*t*|s < 1.61, *p*s > .115, *d*s < 0.19 (Figure 4, orange bars). Still, response costs for repeated negations in the first condition per proportion order differed between groups, with significantly higher response costs for the low-PN-first group relative to the high-PN-first group, *t*(72) = 3.16, *p* = .002, *d* = 0.37.

### Discussion

Experiment 1 revealed negation costs in terms of delayed initiation and execution of responses in negation trials compared to affirmative responses, and a spatial attraction towards the location of the affirmative response in case of negations. Overall, negation costs increased with increasing frequency, replicating previous findings (Gawronski et al., 2008), but they were reduced when an additional factor came into play, namely the recent exposure to a negation operator in the previous trial.^5^ This reduction was particularly pronounced in the AUC measure, and to a lesser extent also in ITs and MTs, in blocks with a current or a previous high frequency of negations.

This finding supports the suggestion that negation effects are not hard-wired, as often assumed, but can indeed be mitigated. Still, even with a majority of negations within a block, their behavioral signature never reversed; although negations were the more frequent response in the high-PN blocks, they were never faster or less attracted to the opposite side than affirmative responses. In line with the *Spinozan model*, these findings suggest that every single activation of a negation requires that its semantic content is initially affirmed, whereas it is negated only in a second step. This second negation step cannot be bypassed by high-frequency training, negations always produce ironic effects (Wegner, 2009). This result also suggests a qualitative difference between the process of negating a response rule on the one hand and consistent ignoring of certain stimuli on the other hand, as training to consistently ignore certain stimuli can reportedly speed up performance (Cunningham & Egeth, 2016). Another interesting finding is that the sequence of conditions that participants experience during the experiment seems to influence how negations are handled. Especially for the response execution, infrequent negations without prior experience (low-PN blocks of the low-PN-first group) barely seem to benefit from recency influences. However, if negations have been experienced on a frequent basis, recency effects emerge, even though the overall costs of negations seem to be unaffected, which speaks against an overall adaptation due to learning and familiarity with the task, but for a specific mechanism that (working memory traces of) recent processing of a negation can now be used to increase future performance.

Before drawing further conclusions from these results, we first present Experiment 2, which was conducted not only to replicate the present results, but also to extend them and to address a potential confound of the experimental design.

## Experiment 2

The results of Experiment 1 showed a consistent reduction of the detrimental effects of negation processing after a recent negation, particularly during or after a context of frequent negations. At the same time, the chosen experimental design comes with two limitations that derive from the experiment’s focus on a limited set of two stimulus-response pairs with rather abstract shape stimuli.

As a first consequence, the processing demands to encode and classify these shape stimuli arguably do not parallel the demands in situations such as person classification that would be involved in negating a stereotype (Gawronski et al., 2008). We therefore opted to use such a person classification task in Experiment 2 and had participants affirm or negate the gender of photographed faces. We again employed a continuous mouse-tracking task, because studies on person construal have shown the corresponding trajectories to be highly sensitive to response biases during gender classification (Freeman, Ambady, Rule, & Johnson, 2008).

As a second, the limited set of two stimulus-response pairs makes it difficult to conclude whether the effects found in Experiment 1 were possibly driven by stimulus repetitions rather than negation repetitions: With only two stimuli, the chance of repeating instruction and stimuli is high, so it might be that if both repeat, responses are not selected anew, but also simply repeated, which might obscure the actual negation response costs. With the person classification task, we could introduce multiple stimuli that had to be classified according to the same rule (male vs. female) to rule out this potential confound.

### Method

A new set of eighty participants was recruited (mean age = 27.4 years, *SD* = 9.0, 19 male, 6 left-handed) that fulfilled the same criteria as in Experiment 1. The data of one participant was removed due to technical difficulties during testing, and two participants were removed from the sample due to high error rates (>25%).

Experiment 2 was similar to Experiment 1 (see Figure 1) with the following changes: We replaced the stimulus shapes that were used to instruct movements to the left or to the right by four pictures of faces (two male, two female, taken from the KDEF picture set, picture codes AF04NES, AF06NES, AM26NES, and AM29NES; Lundqvist, Flykt, & Öhman, 1998; the pictures can be viewed in the Supplementary Material online, page 31). Pictures now had to be categorized as either male or female, and the mapping rule was indicated by a male and female symbol (♂/♀) at the left and right side of the symbol that instructed standard vs. negation responses (!/↺) in between trials (see Figure 1). Gender-response mapping was counterbalanced between participants.

### Results

Data was handled as in Experiment 1. Errors and omissions (6.1%) as well as trials following errors and omissions (5.1%) and outliers (6.0%) were removed. Finally, all stimulus repetitions of the remaining data were removed (19.7%).

Mean ΔITs, ΔMTs, and ΔAUCs were analyzed in a 2 × 2 × 2 ANOVA with preceding response type (standard vs. negation) and proportion negation (low-PN vs. high-PN) as within-subject factors, and proportion order (low-PN-first vs. high-PN-first) as a between-subjects factor. As for Experiment 1, the full analysis of every measure, including the factor current response type, the corresponding raw data, and a separate analysis of the stimulus repetitions can be found in the Supplementary Material online (www.osf.io/kzwp6, page 13 for the full analysis excluding the stimulus repetitions; page 22 for the separate analysis of the stimulus repetitions).

#### ΔITs

**Figure 5:**
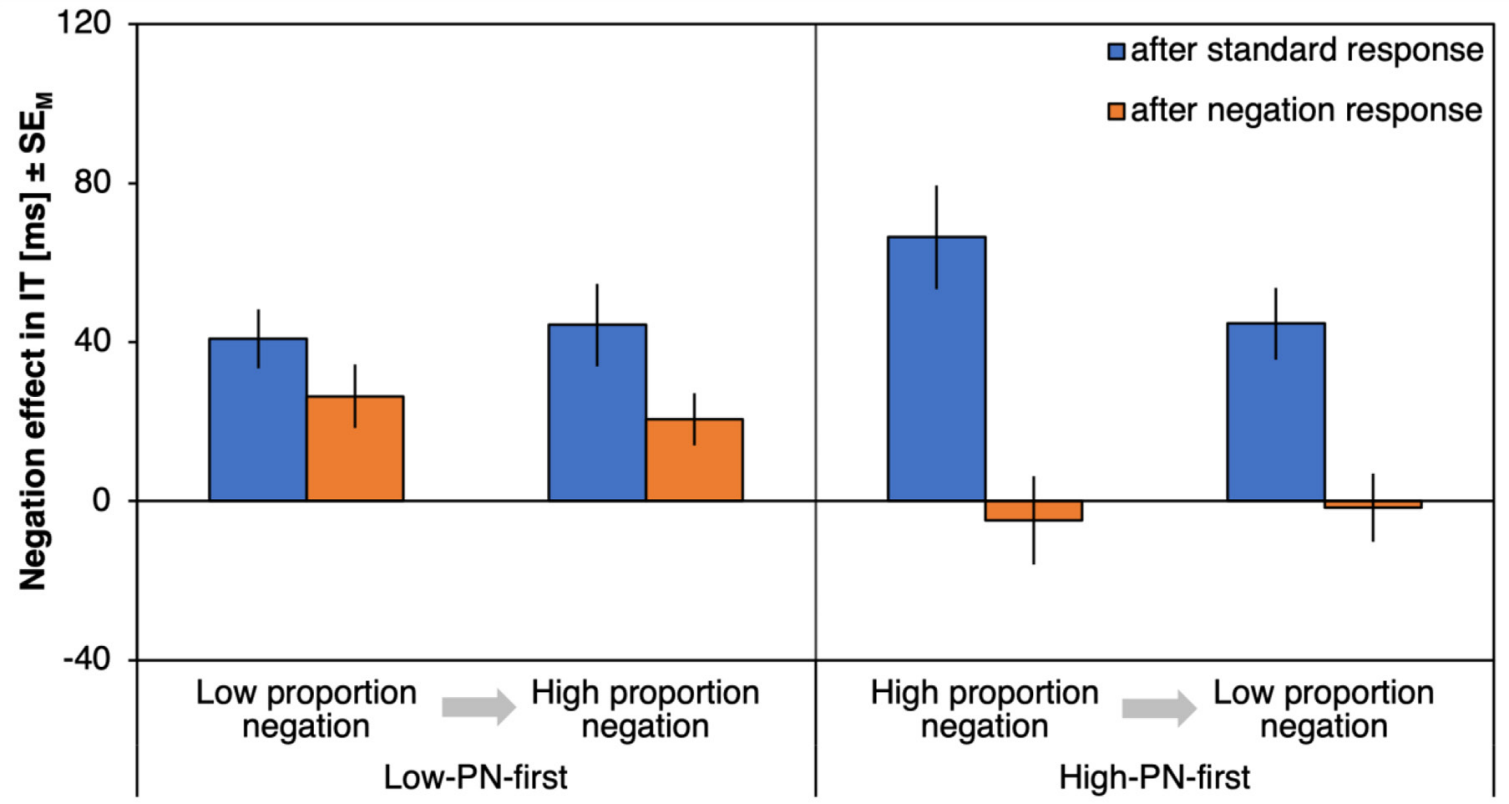
**Results of Experiment 2, initiation times.** Negation effects on initiation times (ΔIT), plotted as a function of proportion negation and proportion order (abscissa) and preceding response type (left, blue bars for trials following standard responses; right, orange bars for trials following negation responses). Error bars represent standard errors of the mean.

Figure 5 shows the negation effects on ITs (ΔIT) as a function of preceding response type, proportion negation and proportion order. There was a significant effect of preceding response type, *F*(1,75) = 42.05, *p* < .001, η_p_^2^ = .36, with stronger negation effects after a standard response (49 ms) compared to after a negation response (10 ms). Negation effects were especially reduced after negation responses (compared to after a standard response) for the high-PN condition (with a reduction of ΔIT by 59 ms) relative to the low-PN condition (with a reduction of 19 ms) as indicated by an interaction of preceding response type and proportion negation, *F*(1,75) = 4.68, *p* = .034, η_p_^2^ = .06. Recency effects differed between groups with the high-PN-first group showing larger differences (59ms) than in the low-PN-first group (19ms), *F*(1,75) = 10.97, *p* = .001, η_p_^2^ = .13. No other effects were significant, *F*s < 1.23, *p*s > .271 (for an overview, see Table 2).

**Table 2:** **Results of Experiment 2.** Overview of the ANOVA results of Experiment 2 for initiation times (ITs), movement times (MTs) and areas under the curve (AUCs). All analyses were run on the negation effects in each design cell (i.e., current negation minus current standard).

	Effect	*F*	*p*	η_p_^2^

**IT**	preceding response type (N-1)	42.05	<.001	.36
	proportion negation (PN)	<1.00	.375	.01
	proportion order (PO)	<1.00	.502	<.01
	N-1 × PN	4.68	.034	.06
	N-1 × PO	10.97	.001	.13
	PN × PO	1.23	.271	.02
	N-1 × PN × PO	<1.00	.336	.01

**MT**	preceding response type (N-1)	102.42	<.001	.58
	proportion negation (PN)	<1.00	.493	.01
	proportion order (PO)	1.64	.204	.02
	N-1 × PN	8.36	.005	.10
	N-1 × PO	6.58	.012	.08
	PN × PO	<1.00	.493	.01
	N-1 × PN × PO	12.95	.001	.15

**AUC**	preceding response type (N-1)	124.53	<.001	.62
	proportion negation (PN)	8.06	.006	.10
	proportion order (PO)	<1.00	.431	.01
	N-1 × PN	20.28	<.001	.21
	N-1 × PO	1.87	.176	.02
	PN × PO	2.16	.146	.03
	N-1 × PN × PO	3.50	.065	.05

Response costs for negations after standard responses differed from zero for all combinations of proportion negation and proportion order, |*t*|s > 4.30, *p*s < .001, *d*s > 0.69 (Figure 5, blue bars), whereas response costs for repeated negations differed from zero only in the low-PN-first subgroup, |*t*|s > 3.12, *p*s < .003, *d*s > 0.50, but not in the high-PN-first subgroup, |*t*|s < 1, *p*s > .671, *d*s < 0.07 (Figure 5, orange bars).

#### ΔMTs

**Figure 6:**
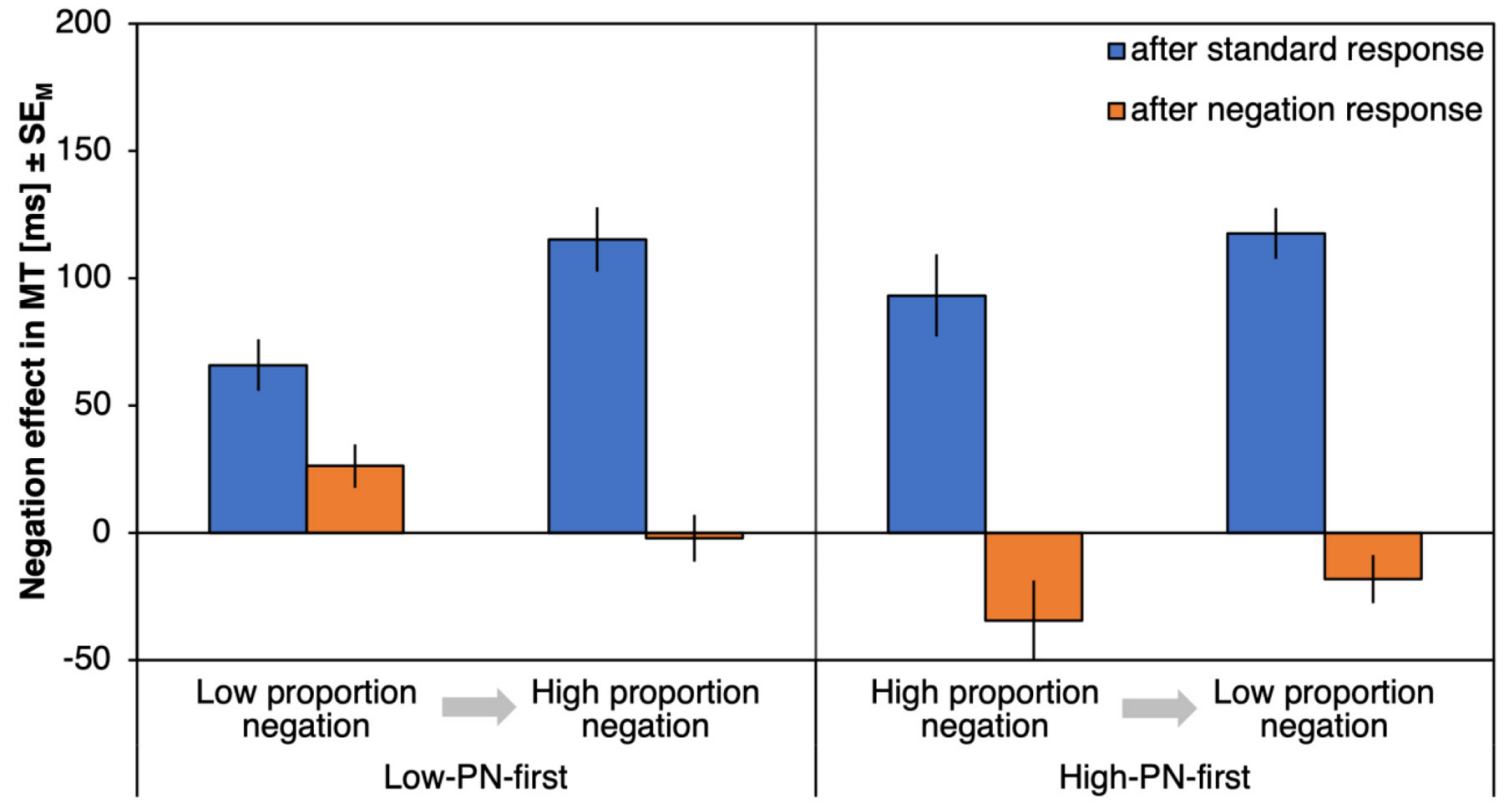
**Results of Experiment 2, movement times.** Negation effects on movement times (ΔMT), plotted as a function of proportion negation and proportion order (abscissa) and preceding response type (left, blue bars for trials following standard responses; right, orange bars for trials following negation responses). Error bars represent standard errors of the mean.

Figure 6 shows the negation effects on MTs (ΔMT) as a function of preceding response type, proportion negation and proportion order. There was a significant effect of preceding response type, *F*(1,75) = 102.42, *p* < .001, η_p_^2^ = .58, with stronger negation effects after a standard response (98 ms) compared to after a negation response (–7 ms). Negation effects were especially reduced after negation responses (compared to after standard responses) for the high-PN condition (with a reduction of ΔMT by 132 ms) relative to the low-PN condition (with a reduction of 78 ms) as indicated by an interaction of preceding response type and proportion negation, *F*(1,75) = 8.36, *p* = .005, η_p_^2^ = .10. Recency effects differed between groups with the high-PN-first group showing larger differences (132 ms) than in the low-PN-first group (78ms), *F*(1,75) = 6.58, *p* = .012, η_p_^2^ = .08. Finally, the three-way interaction was significant, *F*(1,75) = 12.95, *p* = .001, η_p_^2^ = .15, qualifying a much smaller difference between the negation effects after standard and negation responses in the low-PN condition of the low-PN-first subgroup (Figure 6, leftmost bars, Δ = 40 ms) compared to any of the other conditions (Δs > 117 ms), |*t*|*s* > 3.45, *p* < .001, *d* > 0.56, but no other significant differences, |*t*|s > 1, *p*s > .395. No other effects were significant, *F*s < 1.64, *p*s > .204 (for an overview, see Table 2).

Response costs for negations after standard responses differed from zero for all combinations of proportion negation and proportion order, |*t*|s > 5.74, *p*s < .001, *d*s > 0.93 (Figure 6, blue bars), whereas response costs for repeated negations differed from zero only for the first blocks of each PN subgroup, |*t*|s > 2.20, *p*s < .034, *d*s > 0.36, with response benefits for repeated negations in the high-PN blocks of the high-PN-first group, but none of the others, |*t*|s < 1.93, *p*s > .061, *d*s < 0.31 (Figure 6, orange bars).

#### ΔAUCs

**Figure 7:**
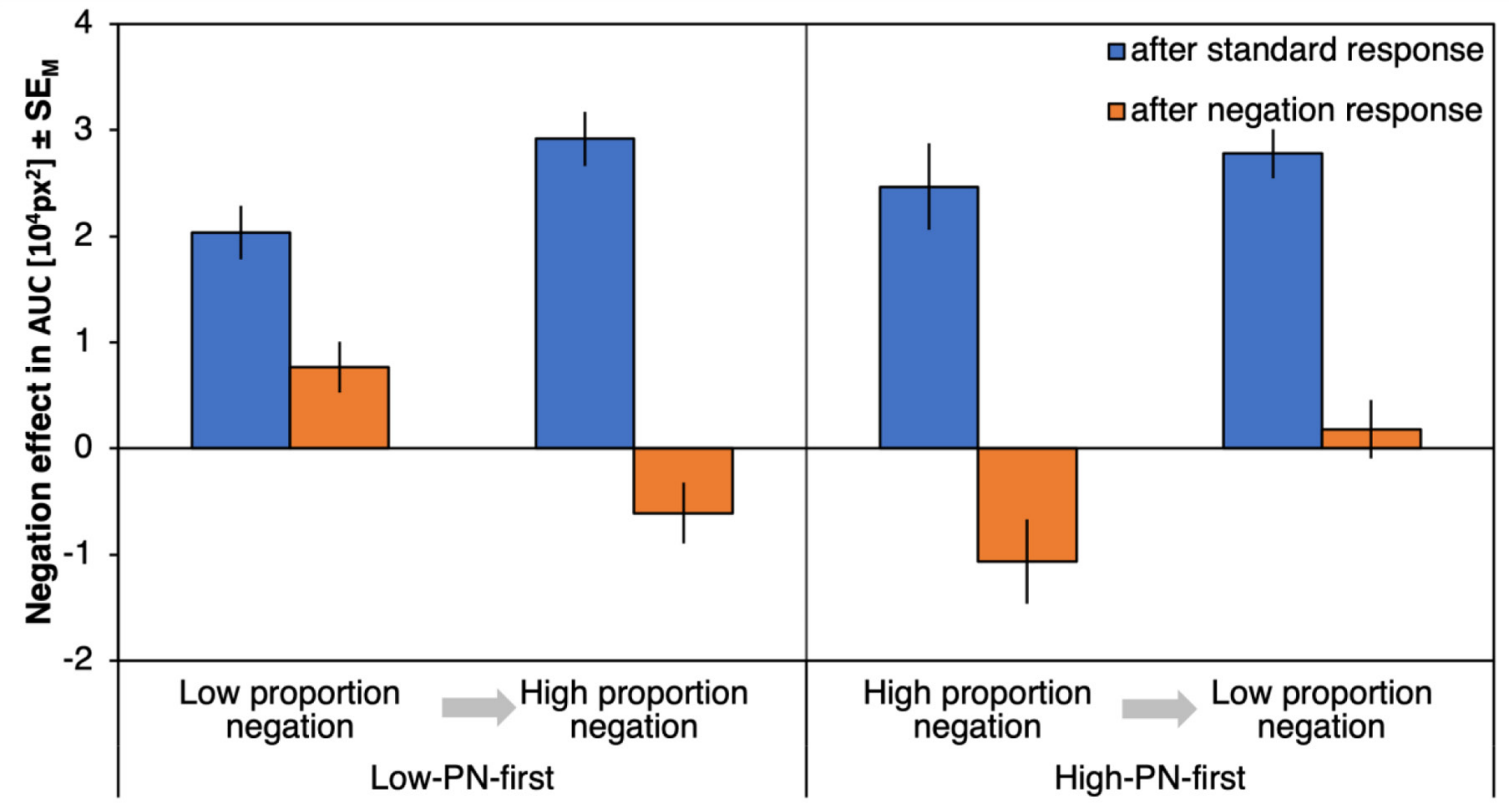
**Results of Experiment 2, areas under the curve.** Negation effects on areas under the curve (ΔAUC), plotted as a function of proportion negation and proportion order (abscissa) and preceding response type (left, blue bars for trials following standard responses; right, orange bars for trials following negation responses). Error bars represent standard errors of the mean.

Figure 7 shows the negation effects on AUCs (ΔAUC) as a function of preceding response type, proportion negation and proportion order. There was a significant effect of preceding response type, *F*(1,75) = 124.53, *p* < .001, η_p_^2^ = .62, with positive negation effects after a standard response (25491 px^2^) and descriptively reversed negation effects after a negation response (–1778 px^2^). Negation effects were overall smaller in the high-PN blocks (10910 px^2^) than in the low-PN blocks (12779 px^2^), *F*(1,75) = 8.06, *p* = .006, η_p_^2^ = .10. Negation effects were especially reduced after negation responses (compared to after standard responses) for the high-PN condition (with a reduction of ΔAUC by 39811 px^2^) relative to the low-PN condition (with a reduction of 27196 px^2^) as indicated by an interaction of preceding response type and proportion negation, *F*(1,75) = 20.28, *p* < .001, η_p_^2^ = .21. No other effects were significant, *F*s < 3.50, *p*s > .065 (for an overview, see Table 2).

Response costs for negations after standard responses differed from zero for all combinations of proportion negation and proportion order, |*t*|s > 6.04, *p*s < .001, *d*s > 0.98 (Figure 7, blue bars), whereas response costs for repeated negations differed from zero for all conditions, |*t*|s > 2.11, *p*s < .042, *d*s > 0.34, with response benefits for repeated negations in the high-PN blocks, except for the low-PN blocks of the high-PN-first subgroup, *t*(37) = 0.66, *p* = .512, *d* = 0.11 (Figure 7, orange bars).

### Discussion

In Experiment 2, we changed the task stimuli and employed a 4:2 mapping rule, where two stimuli (male or female faces) were mapped to each response side. That way, stimulus repetitions could be excluded from the analysis. Still, the results of Experiment 1 could be replicated, with recent negations attenuating negation costs, particularly so, after having experienced a high frequency of negations. Even though the data pattern is overall comparable, there are small differences between the experiments. Experiment 2 produces overall larger negation effects, which might stem from the increased difficulty with four rather than two stimuli. And whereas in Experiment 1 AUCs produced a reliable three-way interaction and MT results were less robust, this seems to be reversed in Experiment 2, with a strong three-way interaction for MTs, but less so for AUCs. It might be that this reflects participants’ priorities, with some focusing more on spatial efficiency when solving the task, and others prioritizing speed. However, this idea requires further research.

Overall, the data pattern of Experiment 2 speaks for the idea that participants can not only benefit from the repetition of specific stimulus-response episodes involving negations (“not triangle”, as in Experiment 1), but that they can also benefit from negations that are less concrete, e.g., negations of the category (“not male”, Experiment 2) or even from repetitions of the negation instruction itself.

Mechanistically, the instruction to affirm or negate a given rule could be construed as two instructional task sets (akin to how telling the truth and lying have been described as intentional task sets, Foerster, Wirth, Kunde, & Pfister, 2017). However, these task sets must come with a hierarchical dependence, as negations (and lies) imperatively require that the original rule (or a representation of the truth) is active in working memory, otherwise there would be nothing to negate (for a similar discussion on the relation between rule-based and rule violation responses, see Wirth et al., 2016, 2018). Switching between these two task sets should then produce switch costs (Monsell, 2003), but the negation task set can never stand on its own without the original semantic content in mind. Therefore, we find a strong switch asymmetry, favoring the switch to the affirmative instruction. The idea of task switching will be further elaborated in the General Discussion.

## General Discussion

In the present experiments, we tested whether the cognitive costs of negation processing can be reduced by a combined manipulation of negation frequency and recency. Previous attempts to reduce the impact of negation processing show that high-frequency training alone does not reduce the impact of negations (Gawronski et al., 2008). Our findings are compatible with this view but, importantly, they show a remarkable effect when frequency and recency manipulations are combined. That is: Negations can indeed be countered effectively, if negation operations are performed frequently and if a particular negation has also been applied very recently. While the impact of recency has largely been neglected in the negation literature (c.f., Wirth et al., 2016), here we describe recency as a crucial factor to mitigate the ironic effects of negations. But only when both, a high frequency and recency, have been experienced, in combination they manage to almost eliminate the burdens of negations. Whereas in conflict tasks, frequency and recency have been suggested to work independently (Funes, Lupiáñez, & Humphreys, 2010), for negations, they might interact (c.f., Wirth et al., 2018): While experiencing (or having experienced) a high frequency seems to signal the necessity for adaptation, recency seems to provide the mechanism for adaptation. This notion is compatible with the *Spinozan approach*, if we assume that negations leave a trace in working memory, and a subsequent negation can profit shortly after from the already negated semantic content, circumventing the cognitively effortful unacceptance of the semantic content for every single instance of a negation.

These results show that negation effects are not as hard-wired as was speculated in the Introduction, rather they can be modulated by exercising cognitive control. This might, in turn, have promising implications for real world scenarios involving the implementation of negated intentions and stereotypes (Gollwitzer & Oettingen, 2012): Planning not to visit Facebook when coursework is due might be detrimental in its first instance, but frequently and repeatedly holding this intention might in fact help the student to a similar degree to specifically intending the desired outcome or training to ignore the tempting cues altogether (Cunningham & Egeth, 2016).

As an alternative explanation for the present results, one might assume that the employed negation task has nothing to do with rule negations per se, but that participants simply construct an opposite rule, and switch between the standard, affirmative rule and the separate, reversed rule. Such spontaneous reversal has been observed in previous work on negation processing (Fillenbaum, 1966; Mayo, Schul, & Burnstein, 2004 for negation in psycholinguistic work; Kawakami, Dovidio, Moll, Hermsen, & Russin, 2000; Gawronski et al., 2008 for the negation of stereotypes). Such an alternative explanation would compromise our interpretation because, if it were the case, then participants should be able to simply switch between those two rules, which should result in faster negation repetitions relative to switches from a negation to a standard response (Monsell, 2003; Rogers & Monsell, 1995). Several observations speak against this possibility, however. In a previous study, we compared a task-switching instruction and a negation instruction directly by having participants either switch between two opposing mapping rules or, alternatively, by having participants respond either according to a standard rule or to negate this rule (Wirth et al., 2016, Exp. 2 and 3). These different instructions resulted in markedly different patterns of results: Whereas the switching instruction yielded robust adaptation effects for all measures, the negation instruction yielded strong negation effects and no signs of adaptation for trajectory measures as outlined in the Introduction. Also speaking against an account in terms of task switching, a full reversal of the negation effect never occurred in either of the present experiments: In Experiment 1, negation effects were present in all conditions, and in Experiment 2, slight reversal of the negation effects occurred, although this reversal was only found in blocks with a high frequency of negations. However, the reversal was not observed systematically, and if observed, this reversal was far from complete, i.e., repeated negations were considerably slower than repeated affirmative responses. Overall, participants seem to readily respond only when the instructed standard rule has to be applied (for more details, see the Supplementary Material online, http://www.osf.io/kzwp6). This is in line with the idea that participants use the rule that they were instructed with, even if more efficient representations might be conceivable (Dreisbach & Haider, 2008, 2009). That said, participants must have some kind of representation of the negated rule in working memory, otherwise there should be no influence of recency (see also Deutsch, Kordts-Freudinger, Gawronski, & Strack, 2009). However, this negated rule is not implemented as a separate task set, but only resides in working memory for some time, and therefore only subsequent responses can be selected based on this short-lived representation, resulting in relatively fast and direct responses only for repeated negations (see Wirth et al., 2016, for a related discussion).

The conceptualization of negations in the present paradigm closely mirrors typical designs that are employed in research on cognitive conflict. This aspect of the experimental design is in line with recent attempts to conceptualize the resolution of negations as conflict processing (de Vega et al., 2016; Dudschig & Kaup, 2018) and it allowed us to access the cognitive architecture underlying negation processing and their mediating processes in a highly controlled setting. How these results translate to more externally valid approaches, e.g., the negation of stereotypes (Gawronski et al., 2008), negation processing involved in lying and dishonesty (Debey, De Hower, & Verschuere, 2014; Foerster, Wirth, Herbort, Kunde, & Pfister, 2017), rule violations (Pfister, Wirth, Schwarz, Steinhauser, & Kunde, 2016; Pfister, Wirth, Schwarz, Foerster, Steinhauser, & Kunde, 2016), or thought suppression (Wegner et al., 1987) still has to be demonstrated. But for now, the combined influence of frequency and recency seems to be the most successful and promising attempt to mitigate ironic negation effects on overt behavior.

## Data Accessibility Statement

The raw data, aggregated data, analytic scripts, and the full results are available at www.osf.io/kzwp6.

## Additional File

The additional file for this article can be found as follows:

10.5334/joc.62.s1 Supplementary Material. Full results, additional analyses, and stimulus material.

## Footnotes

We will review other empirical approaches to negation processing – psycholinguistic approaches (Clark & Chase, 1972, Dudschig & Kaup, 2018), and the negation of S-R rules (Schroder et al., 2008; Wirth et al., 2016) in the introduction to Experiment 1.

Recent psycholinguistic studies have shown that negations affect embodied correlates of language processing such as language-space associations (Dudschig, de la Vega, & Kaup, 2015). Linguistic framing may also be a potent means to diminish negation effects, however, especially via contextual cues how to interpret, construe and imagine the result of a negation (Glenberg, Robertson, Jansen, & Johnson-Glenberg, 1999; Nieuwland & Kuperberg, 2008; Wason, 1965). By contrast, negation costs are readily observed if such contextual cues are not available as in the situation targeted by the present experiments.

More precisely, participants were asked to either affirm or violate a rule (Exp. 1). In this rule violation task, participants behaved similarly to when they were asked to negate a rule (Wirth et al., 2016, Experiment 3), and also this rule violation task produced similar adaptation pattern when the frequency of violations was manipulated (Wirth, Foerster, Herbort, Kunde, & Pfister, 2018).

We removed the first and eleventh block to exclude any practice effects when first starting the experiment, and any relearning and carryover effects when switching PN conditions. But even when these blocks are included, the data pattern does not change in a fundamental way. For the sake of transparency, we provide the raw data and analysis scripts online.

Preceding response type also exerted a strong main effect with negation costs generally being reduced after negation responses as compared to standard responses. This observation mirrors typical findings from the literature on conflict adaptation (e.g., Botvinick et al., 2001; Gratton et al., 1992; Hubbard et al., 2017). However, as for the frequency manipulation, this effect alone was not sufficient to eliminate negation costs consistently.

## Appendix

**Table 3:** Mean values (and SDs) for the negation effect on each dependent variable, separately for each combination of experimental factors, and for each experiment. Initiation time (IT) and movement time (MT) are measured in milliseconds, area under the curve (AUC) is measured in px^2^.

		Low-PN-first group				High-PN-first group			

		Low-PN		High-PN		High-PN		Low-PN	

		After standard	After negation	After standard	After negation	After standard	After negation	After standard	After negation

Experiment 1	ΔIT	45	15	54	19	99	44	41	7
		(44.9)	(47.7)	(64.0)	(46.4)	(106.2)	(56.1)	(62.3)	(26.3)
	ΔMT	72	28	120	35	97	34	72	10
		(59.6)	(47.4)	(76.2)	(55.4)	(69.9)	(40.0)	(76.6)	(32.1)
	ΔAUC	17890	11505	23951	3824	24641	6098	20388	1561
		(15032)	(15452)	(18597)	(14615)	(22020)	(7253)	(18305)	(11135)

Experiment 2	ΔIT	41	26	44	21	66	–5	44	–2
		(46.1)	(49.9)	(64.5)	(41.1)	(80.6)	(68.3)	(55.6)	(53.1)
	ΔMT	66	26	115	–2	93	–34	118	–18
		(63.6)	(53.2)	(78.7)	(57.7)	(100.0)	(96.4)	(61.7)	(58.5)
	ΔAUC	20350	7678	29174	–6090	24681	–10642	27795	1807
		(15873)	(15036)	(15947)	(18036)	(25185)	(24485)	(14152)	(16818)
