# Supplementary material for: How Not to Fall for the White Bear: Combined Frequency and Recency Manipulations Diminish Negation Effects on Overt Behavior
Source: J Cogn. 2019 Apr 23;2(1):11. doi: 10.5334/joc.62 (PMC6634353; doi:10.5334/joc.62)
Supplement: Supplementary Material. — Full results, additional analyses, and stimulus material. [file joc-2-1-62-s1.pdf]

How not to fall for the white bear: Combined frequency and recency manipulations diminish negation effects on overt behavior

- - - Supplementary Material - - -

Robert Wirth, Wilfried Kunde, & Roland Pfister

Department of Psychology

Julius-Maximilians-University of Würzburg

Röntgenring 11

97070 Würzburg

Germany

Corresponding author: Robert Wirth

robert.wirth@uni-wuerzburg.de

phone: +49 - (0)931 - 3181855

Supplementary Material

The following statistics provide a detailed overview of the condensed analyses reported in the article. That is, all following analyses were not computed on the negation effects, but rather on the mean values of each dependent variable for each combination of the factors current response type, preceding response type, and proportion negation.

Experiment 1, full analyses

For the full analyses, after data selection, each measure was analyzed in a separate 2×2×2×2 analysis of variance (ANOVA) with current response type (standard vs. negation), preceding response type, and proportion negation (low-PN vs. high-PN) as within-subject factors, and proportion order (low-PN-first vs. high-PN-first) as a between-subjects factor.

Initiation times.

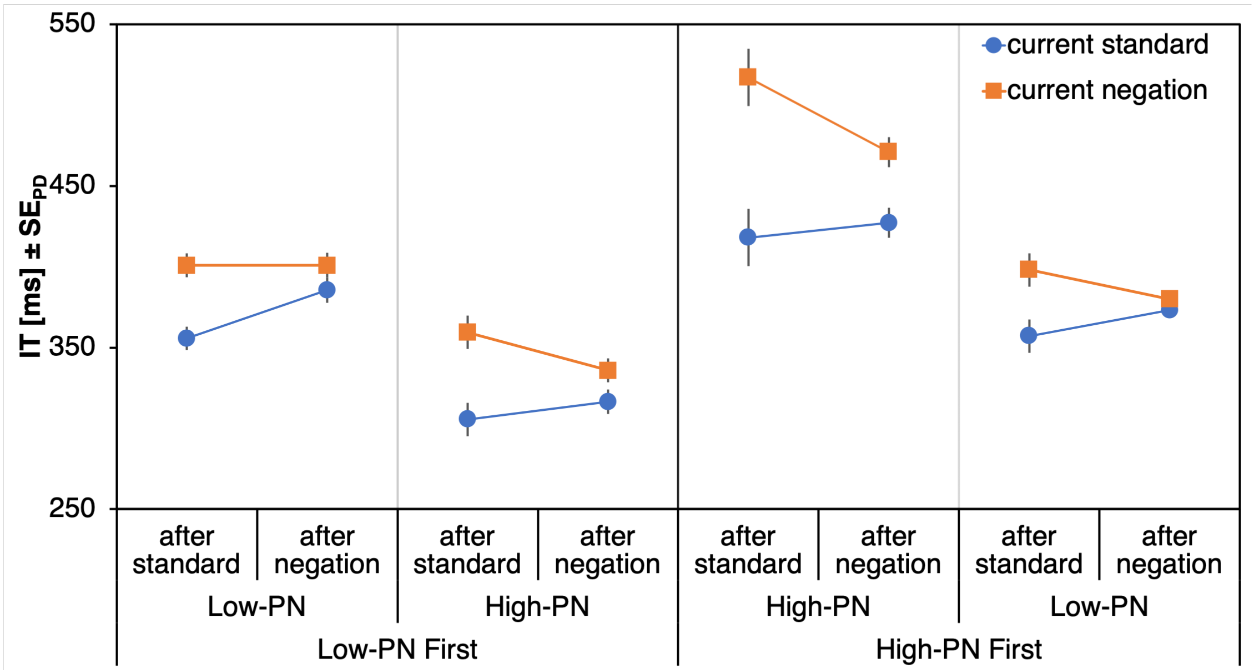

Figure 1. **Full results of Experiment 1, initiation times.** Initiation times (ITs), plotted as a function of proportion negation, proportion order and preceding response type (abscissa) and current response type (blue lines for trials with standard responses; orange lines for trials with negation responses). Error bars represent standard errors of paired differences, computed

separately for each comparison of current standard vs. current negation (Pfister & Janczyk, 2013).

Figure 1 shows the mean ITs as a function of current response type, preceding response type, proportion negation and proportion order. A significant effect of current response type, $F(1,72)=68.54$, $p<.001$, $\eta_p^2=.49$, was driven by faster response initiation for standard responses (367ms) than for negations (407ms). A marginally significant effect of proportion negation, $F(1,72)=2.79$, $p=.099$, $\eta_p^2=.04$, described response initiations in the low-PN condition as faster (382ms) compared to those in the high-PN condition (392ms). Proportion order interacted with proportion negation, $F(1,72)=85.83$, $p<.001$, $\eta_p^2=.54$, with costs for low-PN blocks relative to high-PN blocks for participants who started with the low-PN condition (Δ=-56ms), but benefits for those who started with the high-PN condition (Δ=81ms). An interaction between preceding response type and proportion negation, $F(1,72)=9.02$, $p=.004$, $\eta_p^2=.11$, indicated post-negation slowing in the low-PN condition (Δ=7ms), but post-negation speeding in the high-PN condition (Δ=-12ms). Similarly, there was an interaction between current response type and proportion negation, $F(1,72)=22.34$, $p<.001$, $\eta_p^2=.24$, with a smaller negation effect in low-PN blocks (Δ=27ms) compared to high-PN blocks (Δ=54ms). Also, there was an interaction between current response type and preceding response type, $F(1,72)=24.27$, $p<.001$, $\eta_p^2=.25$, with a stronger negation effect after standard responses (Δ=59ms) than after negation responses (Δ=21ms). There was a three-way interaction between the factors current response type, preceding response type, and proportion negation, $F(1,72)=4.62$, $p=.035$, $\eta_p^2=.06$, as well as an interaction between current response type, proportion negation, and proportion order, $F(1,72)=13.03$, $p=.001$, $\eta_p^2=.15$. None of the remaining effects were significant, *F*s<2.50, *p*s>.118.

To break down this complex pattern of results, i.e., to follow up on the significant higher order interactions, we split the analysis and report the data separately for each proportion order. For this follow-up test we thus conducted two 2×2×2 ANOVAs with current response type (standard vs. negation), preceding response type, and proportion negation (low-PN vs. high-PN) as within-subject factors.

Initiation times, low-PN-first. A significant effect of current response type, $F(1,37)=33.15$, $p<.001$, $\eta_p^2=.47$, was driven by slower response initiations for negations (374ms) than for standard responses (341ms). Response initiation after negation responses was overall slower (360ms) than after standard responses (355ms), $F(1,37)=4.74$, $p=.036$, $\eta_p^2=.11$. Further, response initiation was slower in the low-PN condition (386ms) relative to the high-PN condition (329ms), $F(1,37)=31.39$, $p<.001$, $\eta_p^2=.46$. Response benefits after standard responses emerged for the low-PN condition (Δ=15ms), and response costs emerged for the high-PN condition (Δ=-6ms), as qualified by the interaction of preceding response type and proportion negation, $F(1,37)=16.96$, $p<.001$, $\eta_p^2=.31$. The interaction between preceding response type and current response type was significant, $F(1,37)=11.12$, $p=.002$, $\eta_p^2=.23$, with a stronger effect of negations after standard responses (Δ=50ms) compared to after negation responses (Δ=17ms). Finally, the three-way interaction was not significant, $F<1$, with similar interactions for both, low-PN and high-PN conditions. Current negations did not benefit from previous negations relative to previous standard responses in low-PN blocks (Δ=0ms, $|t|<1$), but they did benefit in the later high-PN blocks (Δ=24ms, $t(37)=3.66$, $p=.001$, $d=0.61$). None of the remaining effects were significant, $Fs<1.14$, $ps>.292$.

Initiation times, high-PN-first. A significant effect of current response type, $F(1,35)=35.63$, $p<.001$, $\eta_p^2=.50$, was driven by slower response initiations for negations (441ms)

than for standard responses (394ms). A significant effect of preceding response type, $F(1,35)=5.06$, $p=.031$, $\eta_p^2=.13$, described responses following standard responses as slower (423ms) compared to responses following negations (413ms). Further, response initiation was slower in the high-PN condition (458ms) relative to the low-PN condition (377ms), $F(1,35)=54.96$, $p<.001$, $\eta_p^2=.61$. Response costs after standard responses emerged for the high-PN condition (Δ=-19ms), but not for the low-PN condition (Δ=-1ms), $F(1,35)=5.10$, $p=.030$, $\eta_p^2=.13$. The interaction between current response type and proportion negation was significant, $F(1,35)=23.10$, $p<.001$, $\eta_p^2=.40$, with a stronger effect of negations in high-PN blocks (Δ=71ms) compared to low-PN blocks (Δ=24ms). The interaction between preceding response type and current response type was significant, $F(1,35)=13.03$, $p=.001$, $\eta_p^2=.27$, with a stronger effect of negations after standard responses (Δ=70ms) compared to after negation responses (Δ=25ms). Finally, the three-way interaction was significant, $F(1,35)=4.86$, $p=.034$, $\eta_p^2=.12$, indicating a significant interaction between preceding and current response type for high-PN blocks, $F(1,35)=8.82$, $p=.005$, $\eta_p^2=.20$, and a significantly stronger interaction for low-PN blocks, $F(1,37)=13.92$, $p=.001$, $\eta_p^2=.29$. Current negations benefitted from previous negations relative to previous standard responses in both, high-PN and low-PN blocks (Δs>18ms, *t*s>1.99, *p*s<.054, *d*s>0.33). None of the remaining effects were significant, *F*s<2.44, *p*s>.127.

Movement times.

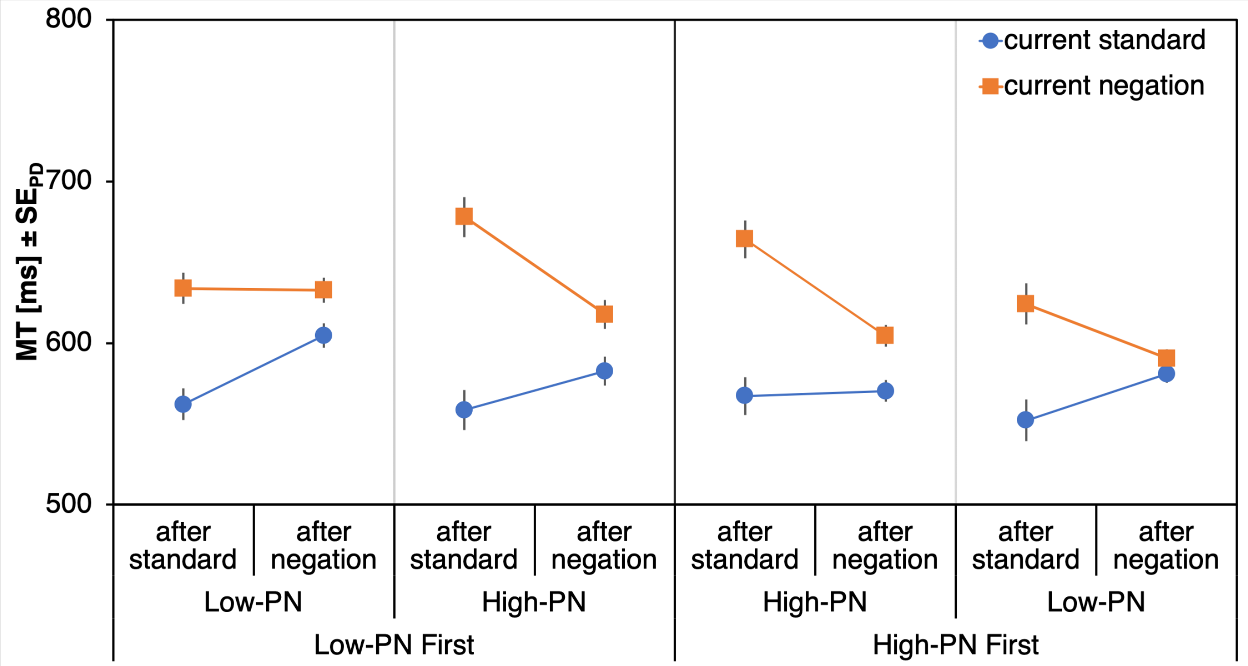

Figure 2. **Full results of Experiment 1, movement times.** Movement times (MTs), plotted as a function of proportion negation, proportion order and preceding response type (abscissa) and current response type (blue lines for trials with standard responses; orange lines for trials with negation responses). Error bars represent standard errors of paired differences, computed separately for each comparison of current standard vs. current negation (Pfister & Janczyk, 2013).

Figure 2 shows the mean MTs as a function of current response type, preceding response type, proportion negation and proportion order. A significant effect of current response type, $F(1,72)=171.68$, $p<.001$, $\eta_p^2=.71$, indicated standard responses (572ms) to be faster than negations (631ms). Responses were slightly faster after negation responses (598ms) relative to after standard responses (605ms), $F(1,72)=6.69$, $p=.012$, $\eta_p^2=.09$. An interaction between preceding response type and proportion order, $F(1,72)=9.55$, $p=.003$, $\eta_p^2=.12$, indicated post-negation speeding in the high-PN-first group (Δ=-15ms), and no difference in the low-PN-first group (Δ=1ms). Current response type interacted with proportion negation, $F(1,72)=24.11$,

$p<.001$, $\eta_p^2=.25$, with a smaller negation effect in low-PN blocks ($\Delta=45$ms) compared to high-PN blocks ($\Delta=72$ms). A similar interaction emerged between preceding response type and proportion negation, $F(1,72)=64.69$, $p<.001$, $\eta_p^2=.47$, with slower responses after negation responses relative to after standard responses in low-PN blocks ($\Delta=9$ms), and the reversed effect in high-PN blocks ($\Delta=-23$ms). Also, there was an interaction between current response type and preceding response type, $F(1,72)=68.33$, $p<.001$, $\eta_p^2=.49$, with a stronger negation effect after standard responses ($\Delta=90$ms) than after negation responses ($\Delta=27$ms). There was a three-way interaction between the factors current response type, preceding response type, and proportion negation, $F(1,72)=4.59$, $p=.035$, $\eta_p^2=.06$, as well as a four-way interaction between all factors, $F(1,72)=4.24$, $p=.043$, $\eta_p^2=.06$. Accordingly, we again conducted follow-up analyses for each proportion order. None of the remaining effects were significant, Fs<2.50, ps$>.118$.

Movement times, low-PN-first. A significant effect of current response type, $F(1,37)=118.58$, $p<.001$, $\eta_p^2=.76$, was driven by slower responses for negations (641ms) than for standard responses (577ms). Response benefits after standard responses emerged for the low-PN condition ($\Delta=21$ms), and response costs emerged for the high-PN condition ($\Delta=-18$ms), $F(1,37)=44.40$, $p<.001$, $\eta_p^2=.55$. The interaction between current response type and proportion negation was also significant, $F(1,37)=9.84$, $p=.003$, $\eta_p^2=.21$, with a stronger effect of negations in high-PN blocks ($\Delta=77$ms) compared to low-PN blocks ($\Delta=50$ms). The interaction between preceding response type and current response type was significant, $F(1,37)=36.19$, $p<.001$, $\eta_p^2=.49$, with a stronger effect of negations after standard responses ($\Delta=96$ms) compared to after negation responses ($\Delta=32$ms). Finally, the three-way interaction between preceding response type, current response type and proportion negation was significant, $F(1,37)=7.02$, $p=.012$, $\eta_p^2=.16$, with a significant interaction between preceding and current response type for high-PN

blocks, $F(1,37)=29.60$, $p<.001$, $\eta_p^2=.44$, and a significantly smaller interaction for low-PN blocks, $F(1,37)=18.16$, $p<.001$, $\eta_p^2=.32$. Especially, current negations did not benefit from pervious negations relative to previous standard responses in low-PN blocks (Δ=1ms, $|t|<1$), but did benefit in the later high-PN blocks (Δ=60ms, $t(37)=5.55$, $p<.001$, $d=0.90$). None of the remaining effects were significant, Fs<1, ps>.696.

Movement times, high-PN-first. A significant effect of current response type, $F(1,35)=61.69$, $p<.001$, $\eta_p^2=.64$, was driven by slower responses for negations (621ms) than for standard responses (568ms). A significant effect of preceding response type, $F(1,35)=13.46$, $p=.001$, $\eta_p^2=.28$, described responses following standard responses as slower (602ms) compared to responses following negations (587ms). The interaction between current response type and proportion negation was significant, $F(1,35)=18.24$, $p<.001$, $\eta_p^2=.34$, with a stronger effect of negations in high-PN blocks (Δ=66ms) compared to low-PN blocks (Δ=41ms). Response costs after standard responses emerged for the high-PN condition (Δ=-28ms), but not for the low-PN condition (Δ=-2ms), $F(1,35)=22.01$, $p<.001$, $\eta_p^2=.39$. The interaction between preceding response type and current response type was significant, $F(1,35)=32.26$, $p<.001$, $\eta_p^2=.48$, with a stronger effect of negations after standard responses (Δ=84ms) compared to after negation responses (Δ=22ms). Finally, the three-way interaction was not significant, $F<1$, with similar interactions for both, low-PN and high-PN conditions. Current negations benefitted from previous negations relative to previous standard responses in both, high-PN and low-PN blocks (Δs>33ms, ts>3.71, ps<.001, ds>0.62). None of the remaining effects were significant, Fs<2.44, ps>.127.

Areas under the curve.

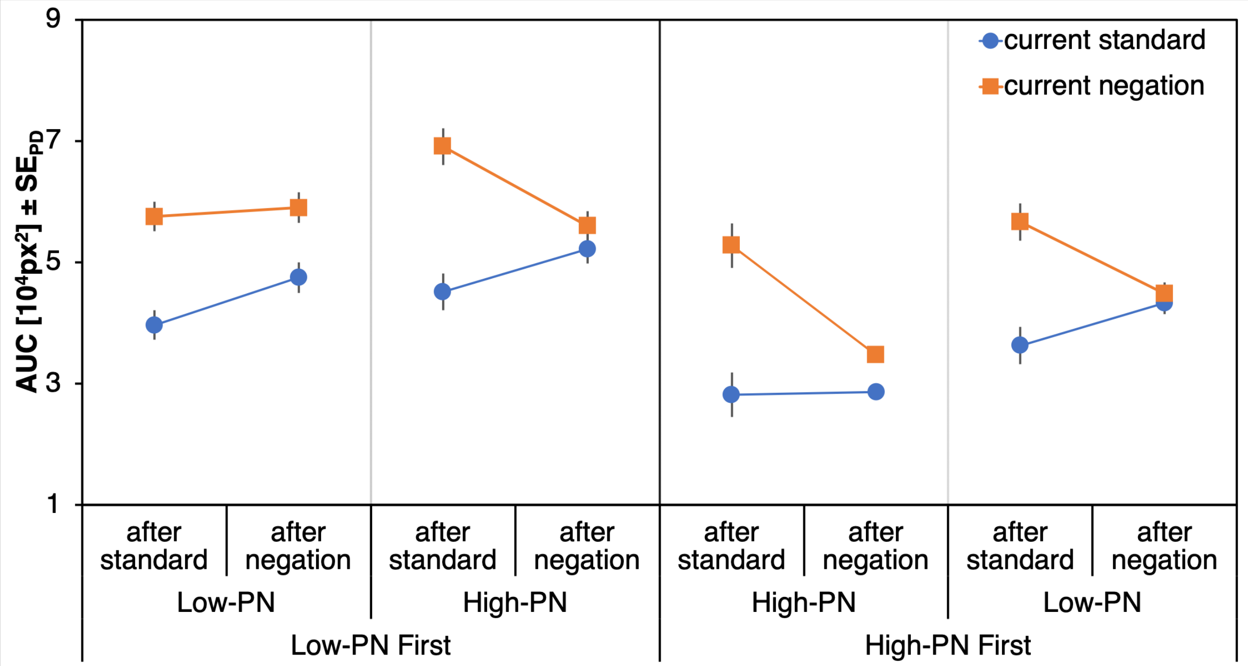

Figure 3. **Full results of Experiment 1, areas under the curve.** Areas under the curve (AUCs), plotted as a function of proportion negation, proportion order and preceding response type (abscissa) and current response type (blue lines for trials with standard responses; orange lines for trials with negation responses). Error bars represent standard errors of paired differences, computed separately for each comparison of current standard vs. current negation (Pfister & Janczyk, 2013).

Figure 3 shows the mean AUCs as a function of current response type, preceding response type, proportion negation and proportion order. A significant effect of current response type, $F(1,72)=135.48$, $p<.001$, $\eta_p^2=.65$, was driven by more curved trajectories for negations (54022px^2) than for standard responses (40274px^2). There was a significant effect of preceding response type, $F(1,72)=11.79$, $p=.001$, $\eta_p^2=.14$, with more curved responses after a standard response (48301px^2) compared to after a negation response (45995px^2). In the high-PN condition, responses were descriptively less curved (46106px^2) than in the low-PN condition (48190px^2), $F(1,72)=3.88$, $p=.053$, $\eta_p^2=.05$. An interaction between preceding response type and

proportion order, *F*(1,72)=21.30, *p*<.001, η_p^2=.23, indicated slight post-negation effects for participants in the low-PN-first group (Δ=823px^2), but a post-negation benefit in the high-PN-first group (Δ=-5610px^2). Proportion order interacted with proportion negation, *F*(1,72)=36.35, *p*<.001, η_p^2=.34, with benefits in low-PN blocks for participants who started with the low-PN condition (Δ=4683px^2) but costs for those who started with the high-PN condition (Δ=-9226px^2). Response benefits after negation responses emerged in the high-PN condition (Δ=-5822px^2) relative to response costs in the low-PN condition (Δ=1208px^2), *F*(1,72)=42.65, *p*<.001, η_p^2=.37. Also, there was an interaction between current response type and preceding response type, *F*(1,72)=53.25, *p*<.001, η_p^2=.43, with a stronger negation effect after standard responses (Δ=21696px^2) than after negation responses (Δ=5799px^2). Finally, there were three-way interactions between the factors current response type, preceding response type, and proportion negation, *F*(1,72)=6.82, *p*=.011, η_p^2=.09, as well as between current response type, proportion negation, and proportion order, *F*(1,72)=4.10, *p*=.047, η_p^2=.05, and a four-way interaction between all factors, *F*(1,72)=7.41, *p*=.008, η_p^2=.09. Accordingly, we again conducted follow-up analyses for each proportion order. None of the remaining effects were significant, *F*s<1.94, *p*s>.168.

Areas under the curve, low-PN-first. A significant effect of current response type, *F*(1,37)=84.22, *p*<.001, η_p^2=.70, was driven by more contorted responses for negations (60430px^2) than for standard responses (46138px^2). Similarly, a significant main effect of proportion negation, *F*(1,37)=7.96, *p*=.008, η_p^2=.18, marked responses in the low-PN-condition as less contorted (50942px^2) than in the high-PN-condition (55626px^2). Response benefits after standard responses emerged for the low-PN condition (Δ=4633px^2), but response costs emerged for the high-PN condition (Δ=-2987px^2), *F*(1,37)=27.72, *p*<.001, η_p^2=.43. The interaction

between preceding response type and current response type was significant, $F(1,37)=19.17$, $p<.001$, $\eta_p^2=.34$, with a stronger effect of negations after standard responses ($\Delta=20921px^2$) compared to after negation responses ($\Delta=7665px^2$). Finally, the three-way interaction between preceding response type, current response type and proportion negation was significant, $F(1,37)=9.79$, $p=.003$, $\eta_p^2=.21$, with a significant interaction between preceding and current response type for high-PN blocks, $F(1,37)=21.86$, $p<.001$, $\eta_p^2=.37$, and a significantly smaller interaction for low-PN blocks, $F(1,37)=4.32$, $p=.045$, $\eta_p^2=.11$. Especially, current negations were descriptively hindered by pervious negations relative to previous standard responses in low-PN blocks ($\Delta=-1440px^2$, $t(37)=-0.64$, $p=.525$, $d=0.10$), but benefit in the later high-PN blocks ($\Delta=13049px^2$, $t(37)=5.29$, $p<.001$, $d=0.86$). None of the remaining effects were significant, $Fs<1.05$, $ps>.313$.

Areas under the curve, high-PN first. A significant effect of current response type, $F(1,35)=54.65$, $p<.001$, $\eta_p^2=.61$, was driven by more contorted responses for negations ($47257px^2$) than for standard responses ($34085px^2$). A significant effect of preceding response type, $F(1,35)=23.69$, $p<.001$, $\eta_p^2=.41$, described responses following standard responses as more contorted ($43476px^2$) compared to responses following negations ($37866px^2$). Similarly, a significant main effect of proportion negation, $F(1,35)=33.40$, $p<.001$, $\eta_p^2=.49$, marked responses in the low-PN-condition as more contorted ($45284px^2$) compared to the high-PN-condition ($36058px^2$). Response costs after standard responses emerged for the low-PN condition ($\Delta=-2407px^2$), but were relatively small compared to the response costs that emerged for the high-PN condition ($\Delta=-8814px^2$), $F(1,35)=16.19$, $p<.001$, $\eta_p^2=.32$. Further, the interaction between current response type and proportion negation was significant, $F(1,35)=5.79$, $p=.022$, $\eta_p^2=.14$, with a stronger effect of negations in high-PN blocks ($\Delta=15370px^2$) compared to low-

PN blocks (Δ=10975px^2). The interaction between preceding response type and current response type was significant, F(1,35)=34.87, p<.001, η_p^2=.50, with a stronger effect of negations after standard responses (Δ=22515px^2) compared to after negation responses (Δ=3830px^2). Finally, the three-way interaction was not significant, F<1, with similar interactions for both, low-PN and high-PN conditions. Current negations benefitted from previous negations relative to previous standard responses in both, high-PN and low-PN blocks (Δs>11819px^2, ts>4.78, ps<.001, ds>0.80).

Experiment 2, full analyses, excluding stimulus repetitions

Again, for the full analyses, after data selection (with all stimulus repetitions removed), each measure was analyzed in a separate 2×2×2×2 ANOVA with current response type (standard vs. negation), preceding response type, and proportion negation (low-PN vs. high-PN) as within-subject factors, and proportion order (low-PN-first vs. high-PN-first) as a between-subjects factor.

Initiation times.

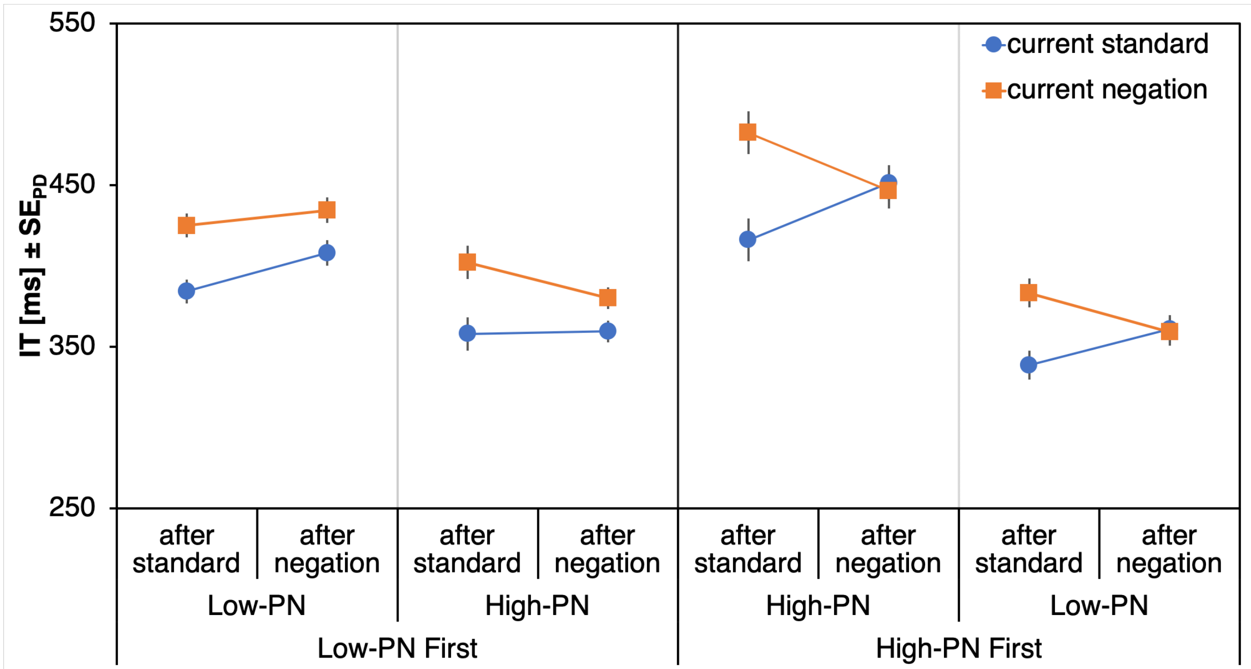

Figure 4. **Full results of Experiment 2 excluding stimulus repetitions, initiation times.** Initiation times (ITs), plotted as a function of proportion negation, proportion order and preceding response type (abscissa) and current response type (blue lines for trials with standard responses; orange lines for trials with negation responses). Error bars represent standard errors of paired differences, computed separately for each comparison of current standard vs. current negation (Pfister & Janczyk, 2013).

Figure 4 shows the mean ITs as a function of current response type, preceding response type, proportion negation and proportion order. A significant effect of current response type, $F(1,75)=33.25$, $p<.001$, $\eta_p^2=.31$, was driven by faster response initiation for standard responses

(385ms) than for negations (414ms). A significant effect of proportion negation, $F(1,75)=10.70$, $p=.002$, $\eta_p^2=.13$, described response initiations in the low-PN condition as faster (387ms) compared to those in the high-PN condition (412ms). Proportion order interacted with proportion negation, $F(1,75)=67.14$, $p<.001$, $\eta_p^2=.47$, with costs for low-PN blocks relative to high-PN blocks for participants who started with the low-PN condition (Δ=-38ms), but benefits for those who started with the high-PN condition (Δ=89ms). An interaction between preceding response type and proportion negation, $F(1,75)=17.58$, $p=.001$, $\eta_p^2=.19$, indicated post-negation slowing in the low-PN condition (Δ=8ms), but post-negation speeding in the high-PN condition (Δ=-5ms). Also, there was an interaction between current response type and preceding response type, $F(1,75)=42.05$, $p<.001$, $\eta_p^2=.36$, with a stronger negation effect after standard responses (Δ=49ms) than after negation responses (Δ=10ms). There was a three-way interaction between the factors current response type, preceding response type, and proportion negation, $F(1,75)=4.68$, $p=.034$, $\eta_p^2=.06$, as well as an interaction between current response type, preceding response type, and proportion order, $F(1,75)=10.97$, $p=.001$, $\eta_p^2=.13$, and an interaction between proportion negation, proportion order, and preceding response type, $F(1,75)=18.74$, $p<.001$, $\eta_p^2=.20$. Accordingly, we again conducted follow-up analyses for each proportion order. None of the remaining effects were significant, *F*s<1.23, *p*s>.271.

Initiation times, low-PN-first. A significant effect of current response type, $F(1,38)=24.98$, $p<.001$, $\eta_p^2=.40$, was driven by slower response initiations for negations (410ms) than for standard responses (377ms). Further, response initiation was faster in the high-PN condition (375ms) relative to the low-PN condition (413ms), $F(1,38)=15.14$, $p<.001$, $\eta_p^2=.29$. Response costs after standard responses emerged for the high-PN condition (Δ=-10ms), but not for the low-PN condition (Δ=17ms), $F(1,38)=26.44$, $p<.001$, $\eta_p^2=.41$. The interaction between preceding

response type and current response type was significant, $F(1,38)=12.05$, $p=.001$, $\eta_p^2=.24$, with a stronger effect of negations after standard responses ($\Delta=43$ms) compared to after negation responses ($\Delta=24$ms). Finally, the three-way interaction was not significant, $F<1$, with similar interactions for both, low-PN and high-PN conditions. Current negations did not benefit from previous negations relative to previous standard responses in low-PN blocks ($\Delta=-9$ms, $t(38)=-1.96$, $p=.057$, $d=0.32$), but they did benefit in the later high-PN blocks ($\Delta=22$ms, $t(38)=4.30$, $p<.001$, $d=0.70$). None of the remaining effects were significant, Fs<1.47, ps>.223.

Initiation times, high-PN-first. A significant effect of current response type, $F(1,37)=11.00$, $p=.002$, $\eta_p^2=.23$, was driven by slower response initiations for negations (418ms) than for standard responses (392ms). Further, response initiation was faster in the low-PN condition (361ms) relative to the high-PN condition (449ms), $F(1,37)=54.34$, $p<.001$, $\eta_p^2=.60$. Also, there was an interaction between current response type and preceding response type, $F(1,37)=29.74$, $p<.001$, $\eta_p^2=.45$, with a stronger negation effect after standard responses ($\Delta=55$ms) than after negation responses ($\Delta=-3$ms). Finally, the three-way interaction between all factors was significant, $F(1,37)=5.06$, $p=.031$, $\eta_p^2=.12$, indicating a significant interaction between preceding and current response type for low-PN blocks, $F(1,37)=19.52$, $p<.001$, $\eta_p^2=.35$, and a significantly stronger interaction for high-PN blocks, $F(1,37)=27.59$, $p<.001$, $\eta_p^2=.43$. Current negations benefitted from previous negations relative to previous standard responses in both PN conditions (Δs>24ms, ts>3.42, ps<.002, ds>0.55. None of the remaining effects were significant, Fs<1.57, ps>.219.

Movement times.

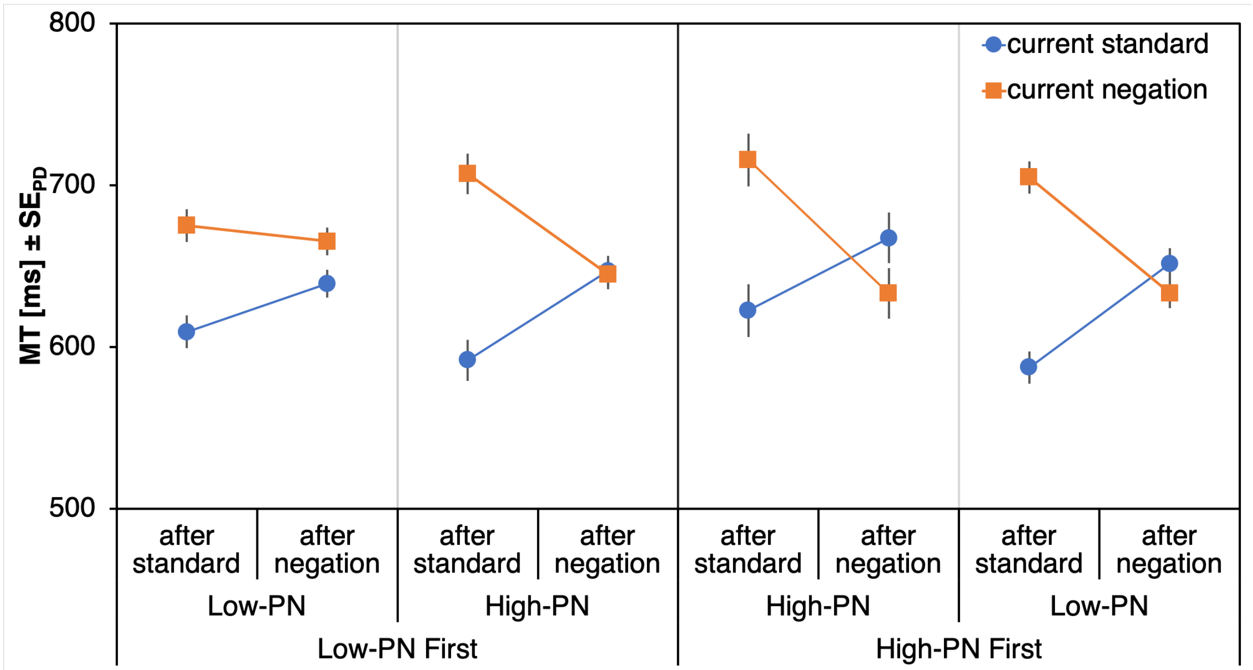

Figure 5. **Full results of Experiment 2 excluding stimulus repetitions, movement times.** Movement times (MTs), plotted as a function of proportion negation, proportion order and preceding response type (abscissa) and current response type (blue lines for trials with standard responses; orange lines for trials with negation responses). Error bars represent standard errors of paired differences, computed separately for each comparison of current standard vs. current negation (Pfister & Janczyk, 2013).

Figure 5 shows the mean MTs as a function of current response type, preceding response type, proportion negation and proportion order. A significant effect of current response type, $F(1,75)=97.99$, $p<.001$, $\eta_p^2=.57$, indicated standard responses (627ms) to be faster than negations (672ms). An interaction emerged between preceding response type and proportion negation, $F(1,75)=6.37$, $p=.014$, $\eta_p^2=.08$, with slower responses after negation responses relative to after standard responses in low-PN blocks (Δ=3ms), and the reversed effect in high-PN blocks (Δ=-11ms). Also, there was an interaction between current response type and preceding response type, $F(1,75)=102.42$, $p<.001$, $\eta_p^2=.58$, with a stronger negation effect after standard responses

(Δ=98ms) than after negation responses (Δ=-7ms). There was a three-way interaction between the factors current response type, preceding response type, and proportion negation, $F(1,75)=8.36$, $p=.005$, $\eta_p^2=.10$, as well as an interaction between current response type, preceding response type, and proportion order, $F(1,75)=6.58$, $p=.012$, $\eta_p^2=.08$, and an interaction between proportion negation, proportion order, and preceding response type, $F(1,75)=4.76$, $p=.032$, $\eta_p^2=.06$, and finally a four-way interaction between all factors, $F(1,75)=12.94$, $p=.001$, $\eta_p^2=.15$. Accordingly, we again conducted follow-up analyses for each proportion order. None of the remaining effects were significant, Fs<1.64, ps>.204.

Movement times, low-PN-first. A significant effect of current response type, $F(1,38)=73.91$, $p<.001$, $\eta_p^2=.66$, was driven by slower responses for negations (673ms) than for standard responses (622ms). Response benefits after standard responses emerged for the low-PN condition (Δ=10ms), and response costs emerged for the high-PN condition (Δ=-3ms), $F(1,38)=4.41$, $p=.042$, $\eta_p^2=.10$. The interaction between preceding response type and current response type was significant, $F(1,38)=37.54$, $p<.001$, $\eta_p^2=.50$, with a stronger effect of negations after standard responses (Δ=91ms) compared to after negation responses (Δ=12ms). Finally, the three-way interaction between preceding response type, current response type and proportion negation was significant, $F(1,38)=38.82$, $p<.001$, $\eta_p^2=.51$, with a significant interaction between preceding and current response type for high-PN blocks, $F(1,38)=53.14$, $p<.001$, $\eta_p^2=.58$, and only a smaller interaction for low-PN blocks, $F(1,38)=10.68$, $p=.002$, $\eta_p^2=.22$. Especially, current negations did not benefit from pervious negations relative to previous standard responses in low-PN blocks (Δ=9ms, $t(38)=1.11$, $p=.273$, $d=0.18$), but did benefit in the later high-PN blocks (Δ=62ms, $t(38)=6.26$, $p<.001$, $d=1.02$). None of the remaining effects were significant, Fs<1.48, ps>.231.

Movement times, high-PN-first. A significant effect of current response type, $F(1,37)=31.96$, $p<.001$, $\eta_p^2=.46$, was driven by slower responses for negations (672ms) than for standard responses (632ms). Further, response execution was faster after negations (646ms) relative to after standard responses (658ms), $F(1,37)=4.82$, $p=.034$, $\eta_p^2=.08$. The interaction between preceding response type and current response type was significant, $F(1,37)=64.26$, $p<.001$, $\eta_p^2=.64$, with a stronger effect of negations after standard responses ($\Delta=105$ms) compared to after negation responses ($\Delta=-26$ms). Finally, the three-way interaction was not significant, $F<1$, with similar interactions for both, low-PN and high-PN conditions. Current negations benefitted from previous negations relative to previous standard responses in both, high-PN and low-PN blocks (Δs>72ms, ts>6.15, ps<.001, ds>0.98). None of the remaining effects were significant, Fs<3.27, ps>.079.

Areas under the curve.

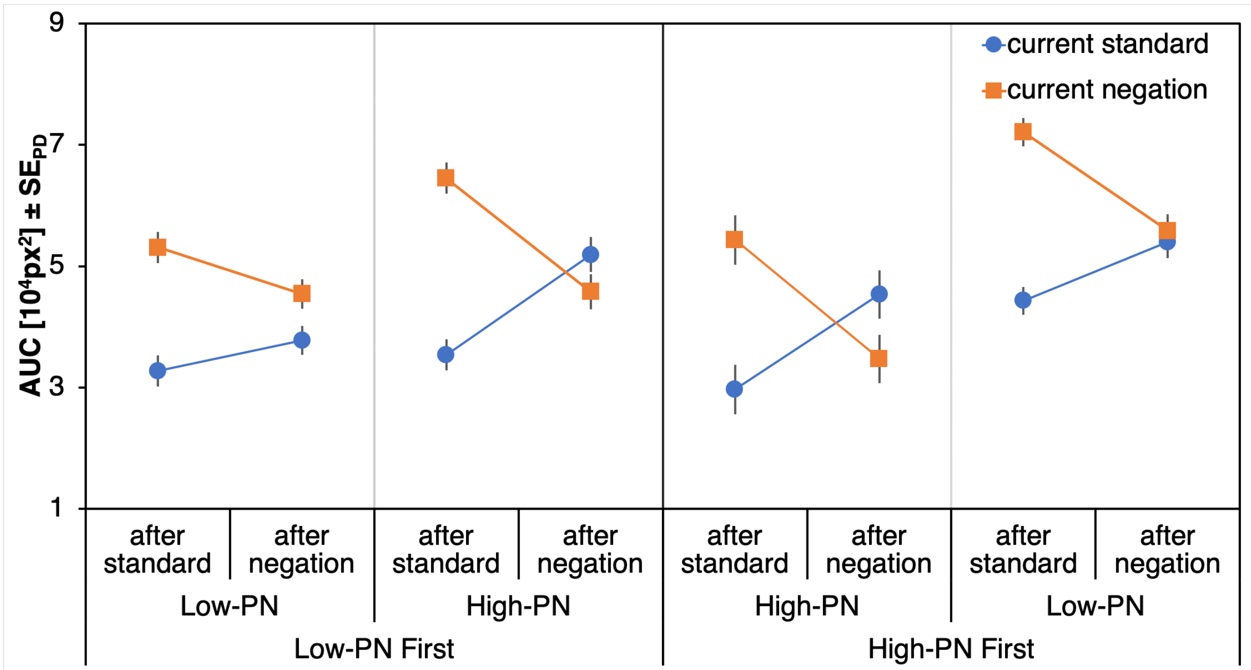

Figure 6. **Full results Experiment 2 excluding stimulus repetitions, areas under the curve.** Areas under the curve (AUCs), plotted as a function of proportion negation, proportion order and preceding response type (abscissa) and current response type (blue lines for trials with standard responses; orange lines for trials with negation responses). Error bars represent standard errors of paired differences, computed separately for each comparison of current standard vs. current negation (Pfister & Janczyk, 2013).

Figure 6 shows the mean AUCs as a function of current response type, preceding response type, proportion negation and proportion order. A significant effect of current response type, $F(1,75)=100.75$, $p<.001$, $\eta_p^2=.57$, was driven by more curved trajectories for negations (54222px^2) than for standard responses (41365px^2). Similarly, responses were more curved after standard responses (46346px^2) compared to after negations (48266px^2), $F(1,75)=6.35$, $p=.014$, $\eta_p^2=.08$. In the high-PN condition, responses were significantly less curved (45231px^2) than in the low-PN condition (49382px^2), $F(1,75)=8.04$, $p=.006$, $\eta_p^2=.10$. Proportion order interacted with proportion negation, $F(1,75)=58.76$, $p<.001$, $\eta_p^2=.44$, with benefits in low-PN blocks for

participants who started with the low-PN condition (Δ=7153px^2) but costs for those who started with the high-PN condition (Δ=-15554px^2). Response costs for negation responses were lower the high-PN condition (Δ=9310px^2) relative to the low-PN condition (Δ=14403px^2), $F(1,75)=8.06$, $p=.006$, $\eta_p^2=.10$. Also, there was an interaction between current response type and preceding response type, $F(1,75)=124.53$, $p<.001$, $\eta_p^2=.62$, with a stronger negation effect after standard responses (Δ=25491px^2) than after negation responses (Δ=-1778px^2). Finally, there were three-way interactions between the factors current response type, preceding response type, and proportion negation, $F(1,75)=20.28$, $p<.001$, $\eta_p^2=.21$, and a four-way interaction between all factors was marginally significant, $F(1,75)=3.50$, $p=.065$, $\eta_p^2=.05$. Accordingly, we again conducted follow-up analyses for each proportion order. None of the remaining effects were significant, *F*s<2.16, *p*s>.146.

Areas under the curve, low-PN-first. A significant effect of current response type, $F(1,38)=68.22$, $p<.001$, $\eta_p^2=.64$, was driven by more contorted responses for negations (52220px^2) than for standard responses (39441px^2). Similarly, a significant main effect of proportion negation, $F(1,38)=19.15$, $p<.001$, $\eta_p^2=.34$, marked responses in the low-PN-condition as less contorted (42254px^2) than in the high-PN-condition (49407px^2). The interaction between preceding response type and current response type was significant, $F(1,38)=61.53$, $p<.001$, $\eta_p^2=.62$, with a stronger effect of negations after standard responses (Δ=24763px^2) compared to after negation responses (Δ=794px^2). Finally, the three-way interaction between preceding response type, current response type and proportion negation was significant, $F(1,38)=38.24$, $p<.001$, $\eta_p^2=.50$, with a significant interaction between preceding and current response type for high-PN blocks, $F(1,38)=77.62$, $p<.001$, $\eta_p^2=.67$, and a significantly smaller interaction for low-PN blocks, $F(1,38)=17.17$, $p<.001$, $\eta_p^2=.31$. Especially, current negations benefitted from

pervious negations relative to previous standard responses in both PN conditions (Δs=7661px^2, *t*s>3.44, *p*s<.001, *d*s>0.56. None of the remaining effects were significant, *F*s<1.82, *p*s>.186.

Areas under the curve, high-PN first. A significant effect of current response type, $F(1,37)=37.25$, $p<.001$, $\eta_p^2=.50$, was driven by more contorted responses for negations (54251px^2) than for standard responses (43340px^2). Also, responses after standard responses were more curved (50113px^2) than after negations (47479px^2), $F(1,37)=4.51$, $p=.041$, $\eta_p^2=.11$. Similarly, a significant main effect of proportion negation, $F(1,37)=39.07$, $p<.001$, $\eta_p^2=.51$, marked responses in the low-PN-condition as more contorted (56573px^2) compared to the high-PN-condition (41019px^2). Response costs for negation responses were higher in the low-PN condition (Δ=14801px^2) than in the high-PN condition (Δ=7019px^2), $F(1,37)=7.34$, $p=.010$, $\eta_p^2=.17$. The interaction between preceding response type and current response type was significant, $F(1,38)=63.68$, $p<.001$, $\eta_p^2=.63$, with a stronger effect of negations after standard responses (Δ=26238px^2) compared to after negation responses (Δ=-4418px^2). Finally, the three-way interaction was not significant, $F(1,38)=2.32$, $p=.136$, $\eta_p^2=.06$, with similar interactions for both, low-PN and high-PN conditions. Current negations benefitted from previous negations relative to previous standard responses in both, high-PN and low-PN blocks (Δs>16265px^2, *t*s>5.24, *p*s<.001, *d*s>0.84). None of the remaining effects were significant, *F*s<1, *p*s>.570.

Experiment 2, full analyses of stimulus repetitions

Again, for the full analyses, after data selection (now only including the stimulus repetitions), each measure was analyzed in a separate 2×2×2×2 ANOVA with current response type (standard vs. negation), preceding response type, and proportion negation (low-PN vs. high-PN) as within-subject factors, and proportion order (low-PN-first vs. high-PN-first) as a between-subjects factor.

Initiation times.

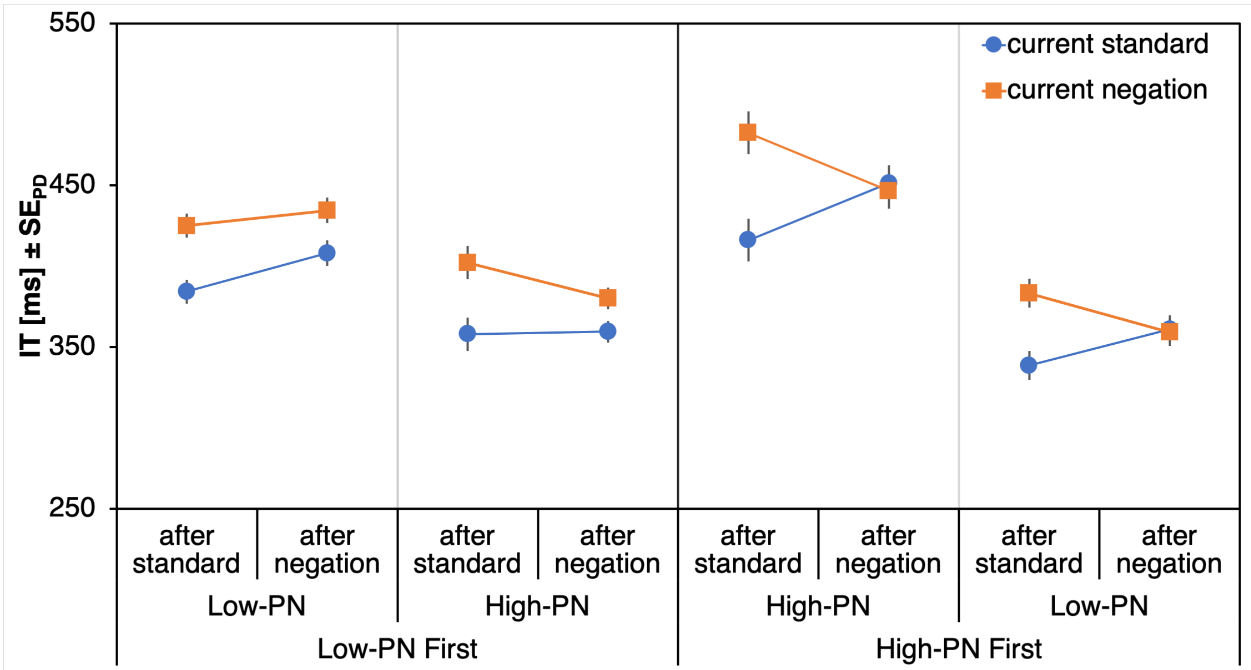

Figure 7. **Full results of stimulus repetitions of Experiment 2, initiation times.** Initiation times (ITs), plotted as a function of proportion negation, proportion order and preceding response type (abscissa) and current response type (blue lines for trials with standard responses; orange lines for trials with negation responses). Error bars represent standard errors of paired differences, computed separately for each comparison of current standard vs. current negation (Pfister & Janczyk, 2013).

Figure 7 shows the mean ITs as a function of current response type, preceding response type, proportion negation and proportion order. A significant effect of current response type, $F(1,75)=31.14$, $p<.001$, $\eta_p^2=.29$, was driven by faster response initiation for standard responses

(375ms) than for negations (401ms). A significant effect of proportion negation, $F(1,75)=7.40$, $p=.008$, $\eta_p^2=.09$, described response initiations in the low-PN condition as faster (378ms) compared to those in the high-PN condition (397ms). Proportion order interacted with proportion negation, $F(1,75)=82.75$, $p<.001$, $\eta_p^2=.53$, with costs for low-PN blocks relative to high-PN blocks for participants who started with the low-PN condition (Δ=-46ms), but benefits for those who started with the high-PN condition (Δ=85ms). An interaction between preceding response type and proportion negation, $F(1,75)=11.05$, $p=.001$, $\eta_p^2=.13$, indicated post-negation slowing in the low-PN condition (Δ=12ms), but post-negation speeding in the high-PN condition (Δ=-8ms). Also, there was an interaction between current response type and preceding response type, $F(1,75)=31.70$, $p<.001$, $\eta_p^2=.30$, with a stronger negation effect after standard responses (Δ=51ms) than after negation responses (Δ=1ms). There was a three-way interaction between the factors current response type, preceding response type, and proportion negation, $F(1,75)=4.29$, $p=.042$, $\eta_p^2=.06$, as well as an interaction between current response type, preceding response type, and proportion order, $F(1,75)=4.09$, $p=.047$, $\eta_p^2=.05$, and a four-way interaction between all factors, $F(1,75)=6.56$, $p=.012$, $\eta_p^2=.08$. Accordingly, we again conducted follow-up analyses for each proportion order. None of the remaining effects were significant, *F*s<1, *p*s>.342.

Initiation times, low-PN-first. A significant effect of current response type, $F(1,38)=21.67$, $p<.001$, $\eta_p^2=.36$, was driven by slower response initiations for negations (397ms) than for standard responses (368ms). Further, response initiation was faster in the high-PN condition (359ms) relative to the low-PN condition (405ms), $F(1,38)=21.67$, $p<.001$, $\eta_p^2=.36$. Response costs after standard responses emerged for the high-PN condition (Δ=-10ms), but not for the low-PN condition (Δ=17ms), $F(1,38)=7.81$, $p=.008$, $\eta_p^2=.17$. The interaction between preceding response type and current response type was significant, $F(1,38)=14.46$, $p=.001$, $\eta_p^2=.28$, with a

stronger effect of negations after standard responses (Δ=45ms) compared to after negation responses (Δ=13ms). Finally, the three-way interaction was not significant, F<1, with similar interactions for both, low-PN and high-PN conditions. Current negations did not benefit from previous negations relative to previous standard responses in low-PN blocks (Δ=1ms, $|t|$<1), but they did benefit in the later high-PN blocks (Δ=25ms, $t(38)=2.71$, $p=.010$, $d=0.43$). None of the remaining effects were significant, Fs<1.84, ps>.183.

Initiation times, high-PN-first. A significant effect of current response type, $F(1,37)=10.89$, $p=.002$, $\eta_p^2=.23$, was driven by slower response initiations for negations (405ms) than for standard responses (382ms). Further, response initiation was faster in the low-PN condition (351ms) relative to the high-PN condition (436ms), $F(1,37)=65.52$, $p<.001$, $\eta_p^2=.64$. Response benefits after standard responses emerged for the low-PN condition (Δ=8ms), and response costs emerged for the high-PN condition (Δ=-7ms), as qualified by the marginally significant interaction of preceding response type and proportion negation, $F(1,37)=3.43$, $p=.072$, $\eta_p^2=.09$. Also, there was an interaction between current response type and preceding response type, $F(1,37)=18.55$, $p<.001$, $\eta_p^2=.33$, with a stronger negation effect after standard responses (Δ=57ms) than after negation responses (Δ=-11ms). Finally, the three-way interaction between all factors was significant, $F(1,37)=9.36$, $p=.004$, $\eta_p^2=.20$, indicating a significant interaction between preceding and current response type for low-PN blocks, $F(1,37)=8.09$, $p=.007$, $\eta_p^2=.18$, and a significantly stronger interaction for high-PN blocks, $F(1,37)=21.62$, $p<.001$, $\eta_p^2=.37$. Current negations did not benefit from previous negations relative to previous standard responses in low-PN blocks (Δ=14ms, $t(37)=1.21$, $p=.234$, $d=0.20$), but they did benefit in the high-PN blocks (Δ=53ms, $t(37)=5.08$, $p<.001$, $d=0.82$). None of the remaining effects were significant, Fs<1.84, ps>.183.

Movement times.

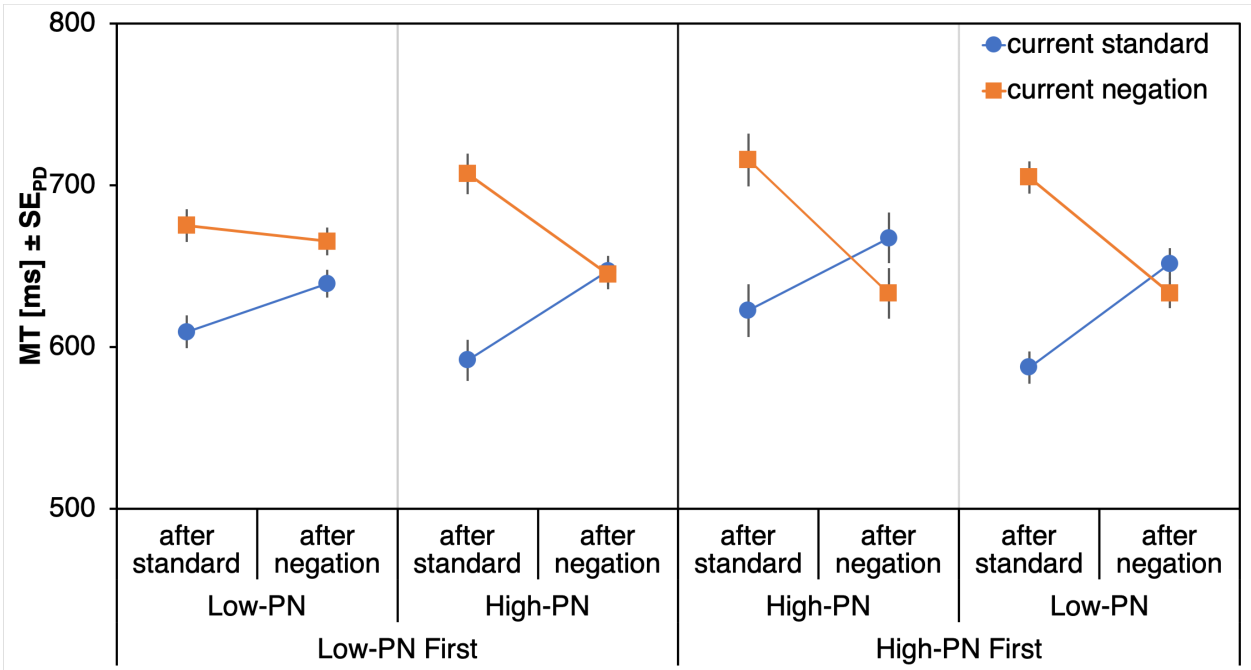

Figure 8. **Full results of stimulus repetitions of Experiment 2, movement times.** Movement times (MTs), plotted as a function of proportion negation, proportion order and preceding response type (abscissa) and current response type (blue lines for trials with standard responses; orange lines for trials with negation responses). Error bars represent standard errors of paired differences, computed separately for each comparison of current standard vs. current negation (Pfister & Janczyk, 2013).

Figure 8 shows the mean MTs as a function of current response type, preceding response type, proportion negation and proportion order. A significant effect of current response type, $F(1,75)=69.29$, $p<.001$, $\eta_p^2=.48$, indicated standard responses (606ms) to be faster than negations (646ms). An interaction emerged between preceding response type and proportion negation, $F(1,75)=16.51$, $p<.001$, $\eta_p^2=.18$, with slower responses after negation responses relative to after standard responses in low-PN blocks (Δ=15ms), and the reversed effect in high-PN blocks (Δ=-14ms). Also, there was an interaction between current response type and preceding response type, $F(1,75)=46.04$, $p<.001$, $\eta_p^2=.38$, with a stronger negation effect after standard responses

(Δ=77ms) than after negation responses (Δ=2ms). There was a three-way interaction between the factors current response type, preceding response type, and proportion negation, $F(1,75)=12.28$, $p=.001$, $\eta_p^2=.14$, as well as an interaction between current response type, preceding response type, and proportion order, $F(1,75)=5.11$, $p=.027$, $\eta_p^2=.06$, and a four-way interaction between all factors, $F(1,75)=7.52$, $p=.008$, $\eta_p^2=.09$. Accordingly, we again conducted follow-up analyses for each proportion order. None of the remaining effects were significant, Fs<1.73, ps>.192.

Movement times, low-PN-first. A significant effect of current response type, $F(1,38)=44.06$, $p<.001$, $\eta_p^2=.54$, was driven by slower responses for negations (652ms) than for standard responses (608ms). Response benefits after standard responses emerged for the low-PN condition (Δ=16ms), and response costs emerged for the high-PN condition (Δ=-10ms), $F(1,38)=8.22$, $p=.007$, $\eta_p^2=.18$. The interaction between preceding response type and current response type was significant, $F(1,38)=13.57$, $p=.001$, $\eta_p^2=.26$, with a stronger effect of negations after standard responses (Δ=69ms) compared to after negation responses (Δ=19ms). Finally, the three-way interaction between preceding response type, current response type and proportion negation was significant, $F(1,38)=21.81$, $p<.001$, $\eta_p^2=.37$, with a significant interaction between preceding and current response type for high-PN blocks, $F(1,38)=31.78$, $p<.001$, $\eta_p^2=.46$, and no interaction for low-PN blocks, $F(1,38)<1$, $p=.998$, $\eta_p^2=.00$. Especially, current negations did not benefit from pervious negations relative to previous standard responses in low-PN blocks (Δ=-16ms, $t(38)=-1.21$, $p=.233$, $d=0.19$), but did benefit in the later high-PN blocks (Δ=60ms, $t(38)=4.59$, $p<.001$, $d=0.73$). None of the remaining effects were significant, Fs<1, ps>.356.

Movement times, high-PN-first. A significant effect of current response type, $F(1,37)=26.42$, $p<.001$, $\eta_p^2=.42$, was driven by slower responses for negations (639ms) than for

standard responses (605ms). Further, response execution was marginally faster in the low-PN condition (613ms) relative to the high-PN condition (631ms), $F(1,37)=3.33$, $p=.076$, $\eta_p^2=.08$. Response costs after standard responses emerged for the high-PN condition ($\Delta=-18$ms), but not for the low-PN condition ($\Delta=13$ms), $F(1,37)=8.34$, $p=.006$, $\eta_p^2=.18$. The interaction between preceding response type and current response type was significant, $F(1,37)=32.51$, $p<.001$, $\eta_p^2=.47$, with a stronger effect of negations after standard responses ($\Delta=85$ms) compared to after negation responses ($\Delta=16$ms). Finally, the three-way interaction was not significant, $F<1$, with similar interactions for both, low-PN and high-PN conditions. Current negations benefitted from previous negations relative to previous standard responses in both, high-PN and low-PN blocks (Δs>34ms, ts>2.44, ps<.020, ds>0.40). None of the remaining effects were significant, Fs<1, ps>.356.

Areas under the curve.

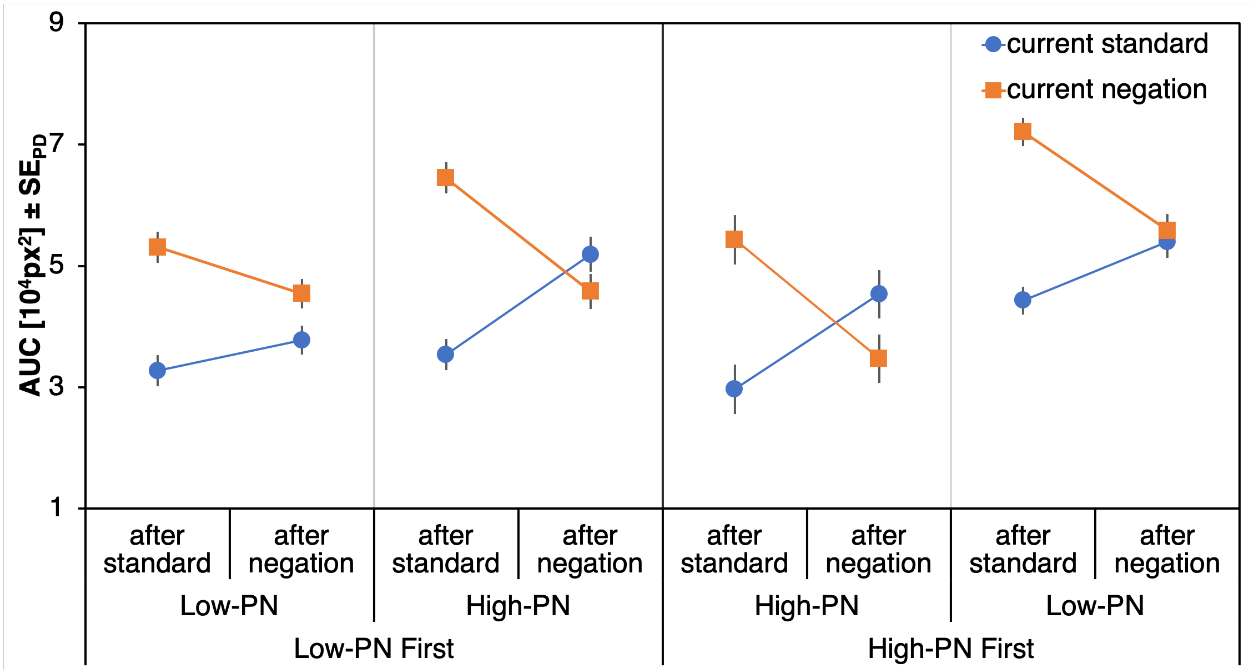

Figure 9. **Full results of stimulus repetitions of Experiment 2, areas under the curve.** Areas under the curve (AUCs), plotted as a function of proportion negation, proportion order and preceding response type (abscissa) and current response type (blue lines for trials with standard responses; orange lines for trials with negation responses). Error bars represent standard errors of paired differences, computed separately for each comparison of current standard vs. current negation (Pfister & Janczyk, 2013).

Figure 9 shows the mean AUCs as a function of current response type, preceding response type, proportion negation and proportion order. A significant effect of current response type, $F(1,75)=97.92$, $p<.001$, $\eta_p^2=.57$, was driven by more curved trajectories for negations (44043px²) than for standard responses (32958px²). In the high-PN condition, responses were significantly less curved (36944px²) than in the low-PN condition (40057px²), $F(1,75)=4.75$, $p=.033$, $\eta_p^2=.06$. A marginally significant interaction between current response type and proportion order, $F(1,75)=3.17$, $p=.079$, $\eta_p^2=.04$, indicated overall positive negation effects for participants in the low-PN-first group ($\Delta=1492$px²), but overall reversed negation effects in the high-PN-first group

(Δ=-1584px^2). Proportion order interacted with proportion negation, $F(1,75)=28.07$, $p<.001$, $\eta_p^2=.27$, with benefits in low-PN blocks for participants who started with the low-PN condition (Δ=4604px^2) but costs for those who started with the high-PN condition (Δ=-11034px^2). Response benefits after negation responses emerged in the high-PN condition (Δ=-5179px^2) relative to response costs in the low-PN condition (Δ=5126px^2), $F(1,75)=26.03$, $p<.001$, $\eta_p^2=.26$. Also, there was an interaction between current response type and preceding response type, $F(1,75)=122.52$, $p<.001$, $\eta_p^2=.62$, with a stronger negation effect after standard responses (Δ=27836px^2) than after negation responses (Δ=-5667px^2). Finally, there were three-way interactions between the factors current response type, preceding response type, and proportion negation, $F(1,75)=9.90$, $p=.002$, $\eta_p^2=.23$, as well as between current response type, preceding response type, and proportion order, $F(1,75)=4.62$, $p=.035$, $\eta_p^2=.06$, and a four-way interaction between all factors, $F(1,75)=10.22$, $p=.002$, $\eta_p^2=.12$. Accordingly, we again conducted follow-up analyses for each proportion order. None of the remaining effects were significant, Fs<1.95, ps>.167.

Areas under the curve, low-PN-first. A significant effect of current response type, $F(1,38)=54.91$, $p<.001$, $\eta_p^2=.59$, was driven by more contorted responses for negations (45688px^2) than for standard responses (32641px^2). Similarly, a significant main effect of proportion negation, $F(1,38)=4.96$, $p=.032$, $\eta_p^2=.12$, marked responses in the low-PN-condition as less contorted (36862px^2) than in the high-PN-condition (41466px^2). Response benefits after standard responses emerged for the low-PN condition (Δ=6785px^2), but response costs emerged for the high-PN condition (Δ=-3802px^2), $F(1,38)=14.99$, $p<.001$, $\eta_p^2=.28$. The interaction between preceding response type and current response type was significant, $F(1,38)=42.87$, $p<.001$, $\eta_p^2=.53$, with a stronger effect of negations after standard responses (Δ=26580px^2)

compared to after negation responses (Δ=-486px^2). Finally, the three-way interaction between preceding response type, current response type and proportion negation was significant, $F(1,38)=17.27$, $p<.001$, $\eta_p^2=.31$, with a significant interaction between preceding and current response type for high-PN blocks, $F(1,38)=68.62$, $p<.001$, $\eta_p^2=.64$, and a significantly smaller interaction for low-PN blocks, $F(1,38)=7.13$, $p=.011$, $\eta_p^2=.16$. Especially, current negations did not benefit from pervious negations relative to previous standard responses in low-PN blocks (Δ=473px^2, $t(38)=0.10$, $p=.923$, $d=0.02$), but did benefit in the later high-PN blocks (Δ=23610px^2, $t(38)=7.15$, $p<.001$, $d=1.14$). None of the remaining effects were significant, Fs<1, ps>.380.

Areas under the curve, high-PN first. A significant effect of current response type, $F(1,37)=44.20$, $p<.001$, $\eta_p^2=.54$, was driven by more contorted responses for negations (42355px^2) than for standard responses (33284px^2). Similarly, a significant main effect of proportion negation, $F(1,37)=27.44$, $p<.001$, $\eta_p^2=.43$, marked responses in the low-PN-condition as more contorted (43337px^2) compared to the high-PN-condition (32303px^2). Response costs after negation responses emerged for the low-PN condition (Δ=3424px^2), but response benefits after negations emerged for the high-PN condition (Δ=-6593px^2), $F(1,37)=11.32$, $p=.002$, $\eta_p^2=.23$. The interaction between preceding response type and current response type was significant, $F(1,38)=81.24$, $p<.001$, $\eta_p^2=.69$, with a stronger effect of negations after standard responses (Δ=29126px^2) compared to after negation responses (Δ=-10985px^2). Finally, the three-way interaction was not significant, $F<1$, with similar interactions for both, low-PN and high-PN conditions. Current negations benefitted from previous negations relative to previous standard responses in both, high-PN and low-PN blocks (Δs>16681px^2, ts>4.17, ps<.001, ds>0.68).

Stimulus Material for Experiment 2 (taken from Lundqvist et al., 1998)

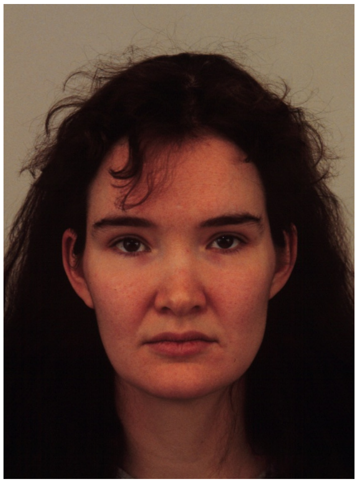

AF04NES

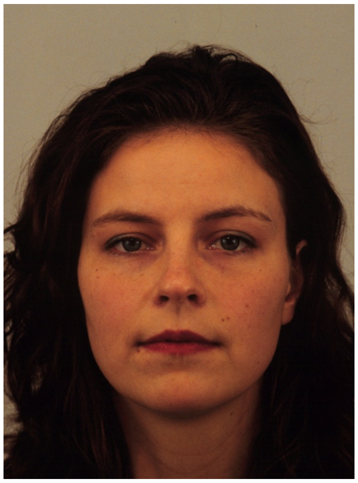

AF06NES

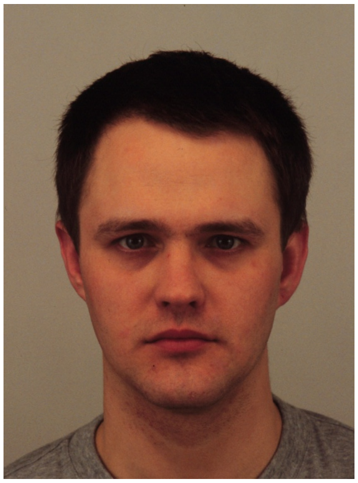

AM26NES

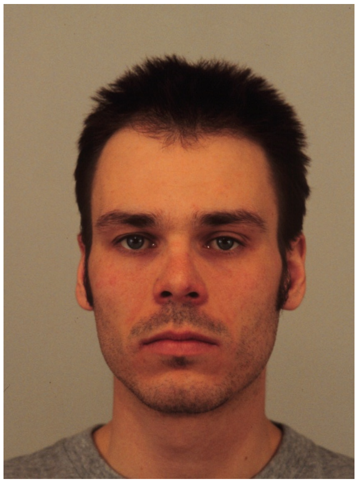

AM29NES

References

Lundqvist, D., Flykt, A., & Öhman, A. (1998). The Karolinska Directed Emotional Faces - KDEF, CD ROM from Department of Clinical Neuroscience, Psychology section, Karolinska Institutet, ISBN 91-630-7164-9.

Pfister, R., & Janczyk, M. (2013). Confidence intervals for two sample means: Calculation, interpretation, and a few simple rules. *Advances in Cognitive Psychology, 9*(2), 74-80.
